# Functional characterization of thermotolerant microbial consortium for lignocellulolytic enzymes with central role of Firmicutes in rice straw depolymerization

**DOI:** 10.1038/s41598-021-82163-x

**Published:** 2021-02-04

**Authors:** Parmeshwar V. Gavande, Arijita Basak, Subhajit Sen, Khusboo Lepcha, Nensina Murmu, Vijeta Rai, Deepika Mazumdar, Shyama Prasad Saha, Vaskar Das, Shilpi Ghosh

**Affiliations:** Department of Biotechnology, University of North Bengal, Raja Rammohunpur, P.O.-NBU, Siliguri, West Bengal 734013 India

**Keywords:** Biotechnology, Microbiology, Environmental sciences

## Abstract

Rice (*Oryza sativa* L.) straw, an agricultural waste of high yield, is a sustainable source of fermentable sugars for biofuel and other chemicals. However, it shows recalcitrance to microbial catalysed depolymerization. We herein describe development of thermotolerant microbial consortium (RSV) from vermicompost with ability to degrade rice straw and analysis of its metagenome for bacterial diversity, and lignocellulolytic carbohydrate active enzymes (CAZymes) and their phylogenetic affiliations. RSV secretome exhibited cellulases and hemicellulases with higher activity at 60 °C. It catalysed depolymerization of chemical pretreated rice straw as revealed by scanning electron microscopy and saccharification yield of 460 mg g^−1^ rice straw. Microbial diversity of RSV was distinct from other compost habitats, with predominance of members of phyla Firmicutes, Proteobacteria and Bacteroidetes; and *Pseudoclostridium*, *Thermoanaerobacterium*, *Chelatococcus* and *Algoriphagus* being most abundant genera. RSV harboured 1389 CAZyme encoding ORFs of glycoside hydrolase, carbohydrate esterase, glycosyl transferase, carbohydrate binding module and auxiliary activity functions. Microorganisms of Firmicutes showed central role in lignocellulose deconstruction with importance in hemicellulose degradation; whereas representatives of Proteobacteria and Bacteroidetes contributed to cellulose and lignin degradation, respectively. RSV consortium could be a resource for mining thermotolerant cellulolytic bacteria or enzymes and studying their synergism in deconstruction of chemically pretreated rice straw.

## Introduction

Plant lignocellulose biomass has been recognized as an abundant and sustainable source of fermentable sugars for production of biofuel and other chemicals^1,2^. Rice straw is a readily available lignocellulose residue with global production of 731 million tons per year. The disposal of underutilized rice straw by burning in the fields deteriorates the ecosystem affecting human health. In rice straw the polymers of cellulose (40%), hemicellulose (35%), lignin (10%), and silica (5%) are crosslinked to form rigid structure and therefore, it shows relatively more resistance to microbial degradation^3,4^. Hence, the development of efficient and cost effective approaches are essential for high value utilization potential of rice straw as bioresource.

Carbohydrate active enzymes (CAZymes) are involved in building and breakdown of complex carbohydrates and glycoconjugates for several biological processes. They are grouped into various families, like glycoside hydrolase (GH), glycosyl transferase (GT), carbohydrate binding module (CBM), carbohydrate esterase (CE), auxiliary activities (AA) and polysaccharide lyase (PL)^2,5^. Among these cellulolytic GHs like, cellulases and hemicellulases play important role in cellulosic depolymerisation; CBMs are required for binding of cellulolytic enzymes to their substrates; AAs are associated with degradation of lignin polymers; and CEs are keys to effective hemicellulase activity^5,6^.

Microorganisms in natural lignocellulose degradation environments have evolved distinct enzyme systems with specific catalytic strategies for depolymerization of cellulosic substrates. For example, aerobic organisms, like *Trichoderma reesei* and actinomycetes produce and secrete free enzymes, whereas anaerobic bacteria, such as *Clostridium thermocellum* and *Acetivibrio cellulolyticus,* integrate various cellulases and xylanases into large multi-enzyme complex called ‘cellulosome’^7,8^. However, cellulolytic system of individual strain often lacks one or more enzyme(s) leading to inefficient cellulose depolymerisation, and also, such system might undergo metabolic repression^7,8^. On the other hand, lignocellulose degradation in natural ecosystem is highly efficient due to the synergistic action of multiple enzymes produced by taxonomically distinct microorganisms. The composting habitat involves aerobic and anaerobic degradation of the plant biomass at high solid state by indigenous microbes and hence, it could be a promising resource of lignocellulose recycling enzymes^1,2^. Vermicomposting is a simple biotechnological process of composting with the aid of earthworms (*Eisenia fetida*). The process is faster than composting because the plant materials pass through the earthworm gut, whereby the resulting earthworm casting has rich microbial load. Also, the worm casting contains higher percentage of macro- and micro-nutrients supporting microbial diversity to a greater extent^9^. However, vermicompost has never been explored for lignocellulose degradation capacity.

In recent years, the metagenomic approach for identification of metabolic functions of multispecies consortia has progressed considerably. Metagenomic analyses of natural cellulose degrading ecosystems have led to the identification of their microbial diversity and cellulolytic genes. The enzymes encoded by the cellulolytic genes have been used for comprehensive understanding of cellulose degradation process^1,2^. The approach is also beneficial for mining genes encoding enzymes with novel properties, like improved tolerance to salt, high temperature and ionic liquid. However, the natural ecosystems are often very complex for direct identification of microorganisms and enzymes involved in cellulose deconstruction. Enrichment culture with lignocellulosic biomaterial as sole carbon source can generate microbial communities with improved capacity to depolymerize plant cellulosic materials^1,10^. Although it can not be directly used for controlled industrial degradation processes due to their dynamic nature and inherent complexity, they can serve as resource for lesser complex and effective microbial consortia with improved application in controlled industrial processes^11^.

The process to access lignocellulosic sugars requires pretreatment for removal of lignin followed by digestion of cellulose components by a cocktail of cellulolytic enzymes. Various physical, chemical and biological pretreatments have been applied, including, hydrothermal, mild acid, mild alkali, ammonia fiber expansion (AFEX), and ionic-liquid pretreatments. An ideal pretreatment should be cheap, removes as much as lignin, maximizes the utilization of lignocellulose with minimal glucan loss, and less inhibitor generation^12,13^. Furthermore, cellulosic degradation by thermotolerant microorganism is advantageous because they produce enzymes with higher temperature optima, at which the cell wall get disorganized supporting better penetration capacity of the enzymes to crystalline cellulose^14^. In addition, thermostable enzymes have higher specific activity and stability; and they allow more flexibility to process configuration; leading to overall economy of the process^15^.

The process of vermicomposting involves a stage at higher temperatures (50 to 55 °C) and hence, vermicompost naturally contains several thermotolerant microorganisms^9^. Present study describes the development of a thermotolerant microbial consortium (RSV) from vermicompost with the ability to degrade rice straw and analysis of its metagenome for bacterial diversity, and lignocellulolytic carbohydrate active enzymes (CAZymes) and their phylogenetic affiliation; and thus providing an insight into the role of different microbes and their enzymes in lignocellulose biomass degradation. RSV can be used for isolation of cellulolytic bacteria or enzymes and studying their synergism in deconstruction of chemically pretreated rice straw.

## Results and discussion

### Development of thermotolerant rice straw degrading microbial (RSV) consortium

**Figure 1:**
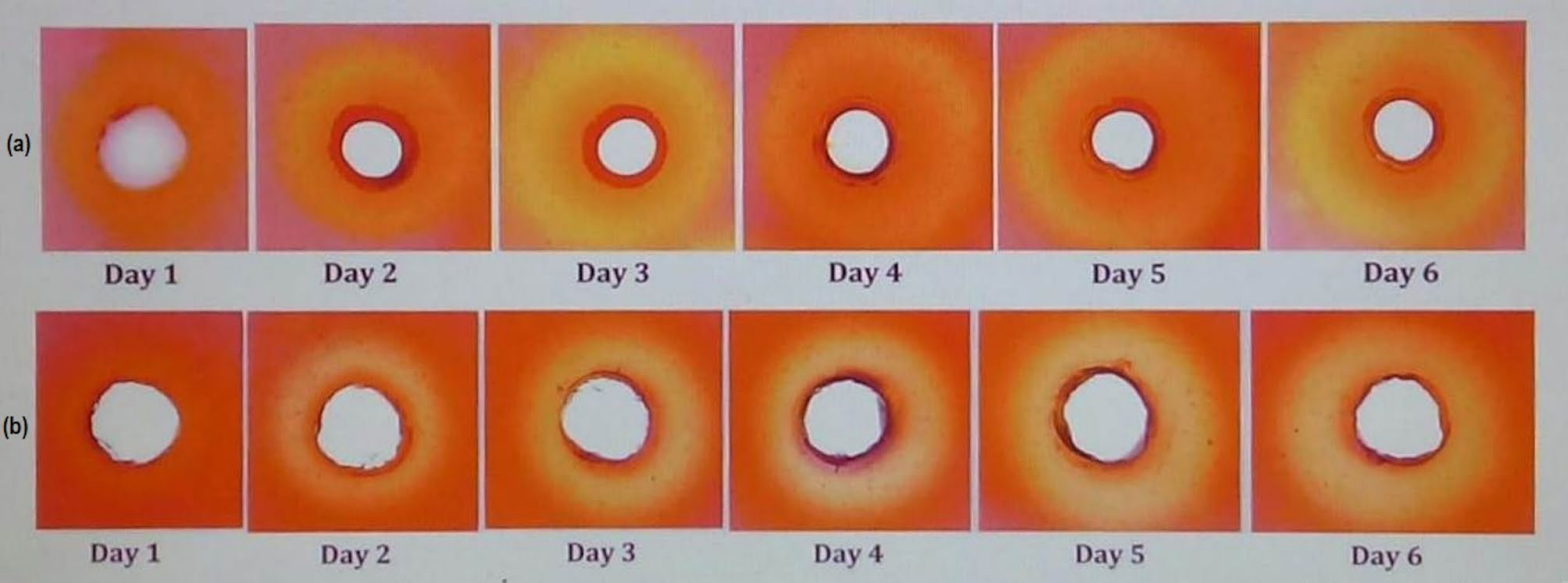
Detection of biodegradation of (**a**) CM-cellulose and (**b**) xylan by RSV consortium at various days of growth, as determined by agarose well diffusion assay.

The cellulosic components of rice straw are relatively more resistant to microbial catalyzed depolymerization primarily due to the presence of relatively higher silica and ash contents^4^. Microbial consortium adapted to grow in a medium with rice straw as sole carbon source at higher temperature, can express multiple enzyme systems with higher activity and stability, whose synergistic action can be an efficient approach for biodegradation of rice straw to fermentable sugars^1,10^. In our study, microbial community of vermicompost was adapted to grow in M9 minimal medium supplemented with rice straw (3% w/v) at 60 °C for 10 weeks to generate a thermotolerant consortium (RSV) for depolymerization of cellulosic polymers of rice straw. In previous studies, adaptive cultivation of the microbial communities of the lignocellulose degradation environments, like compost^1,2,10^, bovine rumen^16^, and termite gut^17^, in culture media with lignocellulosic materials as sole carbon source have been used for developing simplified consortia for depolymerization of specific feedstock. In preliminary experiments, the cellulose degradation ability of RSV consortium was ascertained by agarose well diffusion assay of xylanase and cellulase for six consecutive days of growth, using the tenth passage culture. The cell free supernatant (CFS) of RSV culture formed hydrolytic zones in xylan and cellulose plates indicating the ability of the consortium to produce xylanases and cellulases, respectively. The hydrolysis of cellulose was detectable after day 1, whereas significant xylan hydrolysis was noted after day 2 of incubation (Fig. 1).

### Dynamics of xylanases and cellulases activities of RSV secretome

**Figure 2:**
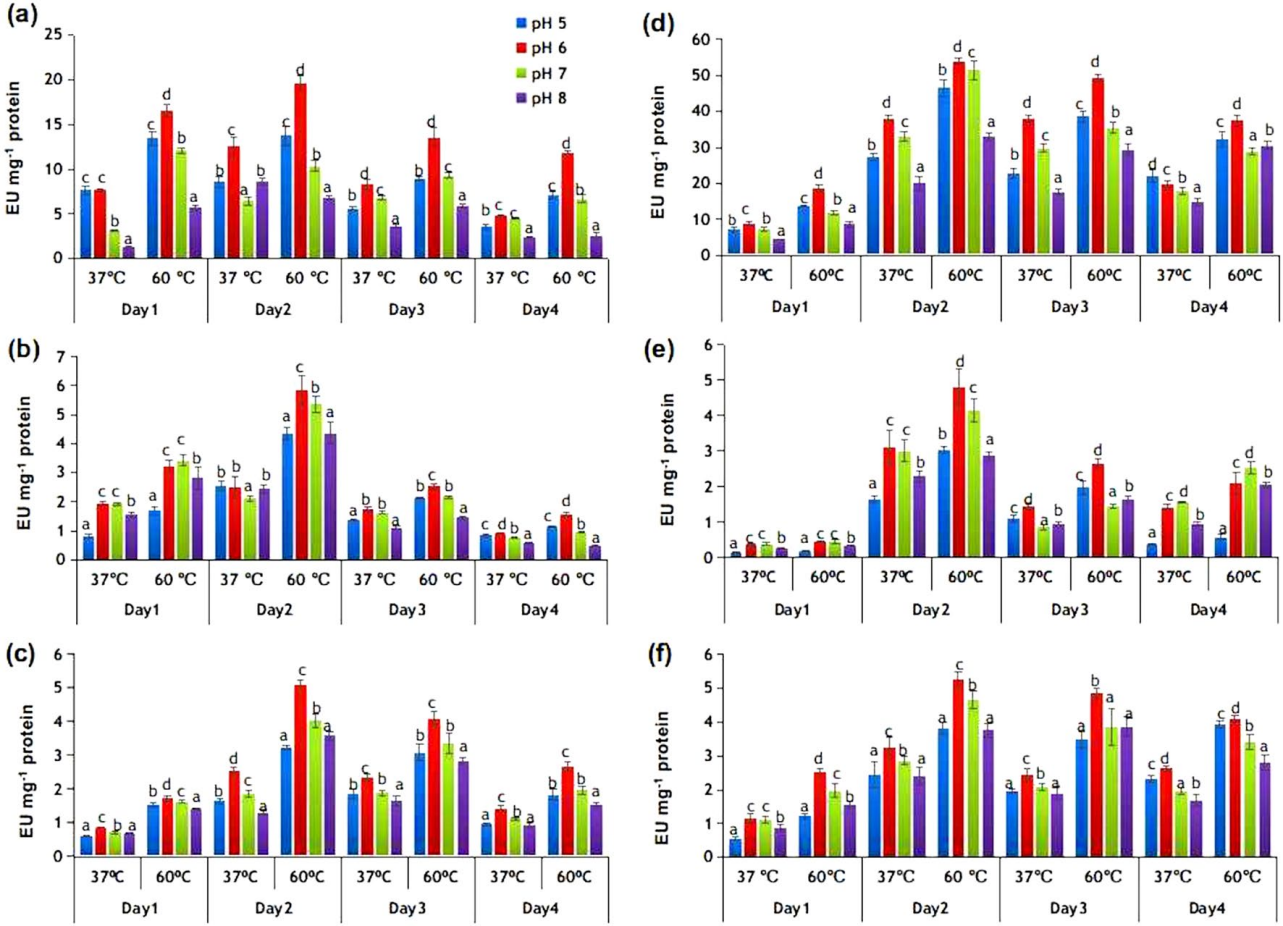
Glycoside hydrolase activities in cell free supernatant of RSV consortium: effect of temperature and pH on activities of (**a**) endoglucanase, (**b**) cellobiohydrolase, (**c**) β-glucosidase, (**d**) endoxylanase, (**e**) β-xylosidase, and (**f**) arabinofuranosidase, during four days of growth of RSV. Data marked with different letters are significantly different (p < 0.05) as suggested by one-way ANOVA followed by DUNCAN multiple range test.

RSV was further analyzed for activities of various glycoside hydrolases (GHs) belonging to xylanases, like endoxylanase, β-xylosidase and α-arabinofuranosidase; and celullases, like endoglucanase, exoglucanase (cellobiohydrolase) and β-glucosidase (Fig. 2). All the enzymatic activities were determined in the pH range 5 to 8 and at two different temperatures (37 and 60 °C) for four consecutive days of growth, using the CFS of tenth passage culture. The results in Fig. 2 reveal that all the enzymes had significantly greater activity at 60 °C, suggesting thermotolerant nature of the RSV enzyme systems. Among the cellulases, the activity of endoglucanase appeared after day 1 of incubation. Although the enzyme showed maximum activity at pH 6 and day 2, it maintained substantial activity till day 4 of incubation (Fig. 2a). Similarly, the activity of exoglucanase or cellobiohydrolase appeared after day1, increased by almost twofold after day 2 and then lowered (Fig. 2b). The activity of β-glucosidase appeared after day 1 and became maximum after day 2. The enzyme exhibited significantly higher activity at all the tested pH with pH 6 being the optimum pH (Fig. 2c). Among xylanases, the activity of endoxylanase was considerably higher than that of β-xylosidase and α-L-arabinofuranosidase. Endoxylanase activity was obtained at all the tested pH with greater activity at pH 6 and was relatively maintained throughout the experimental period (Fig. 2d). Similarly, β-xylosidase and α-L-arabinofuranosidase activities were obtained during all four days and were relatively higher at pH 6 and 7 (Fig. 2e,f). The results thus suggest that the RSV consortium on adaptation to grow at 60 °C produced moderately thermostable GHs for the hydrolysis of cellulosic components of rice straw and all the tested enzymes had significantly greater activity after day 2 of incubation (P < 0.05).

### Saccharification of pretreated rice straw by RSV secretome

**Figure 3:**
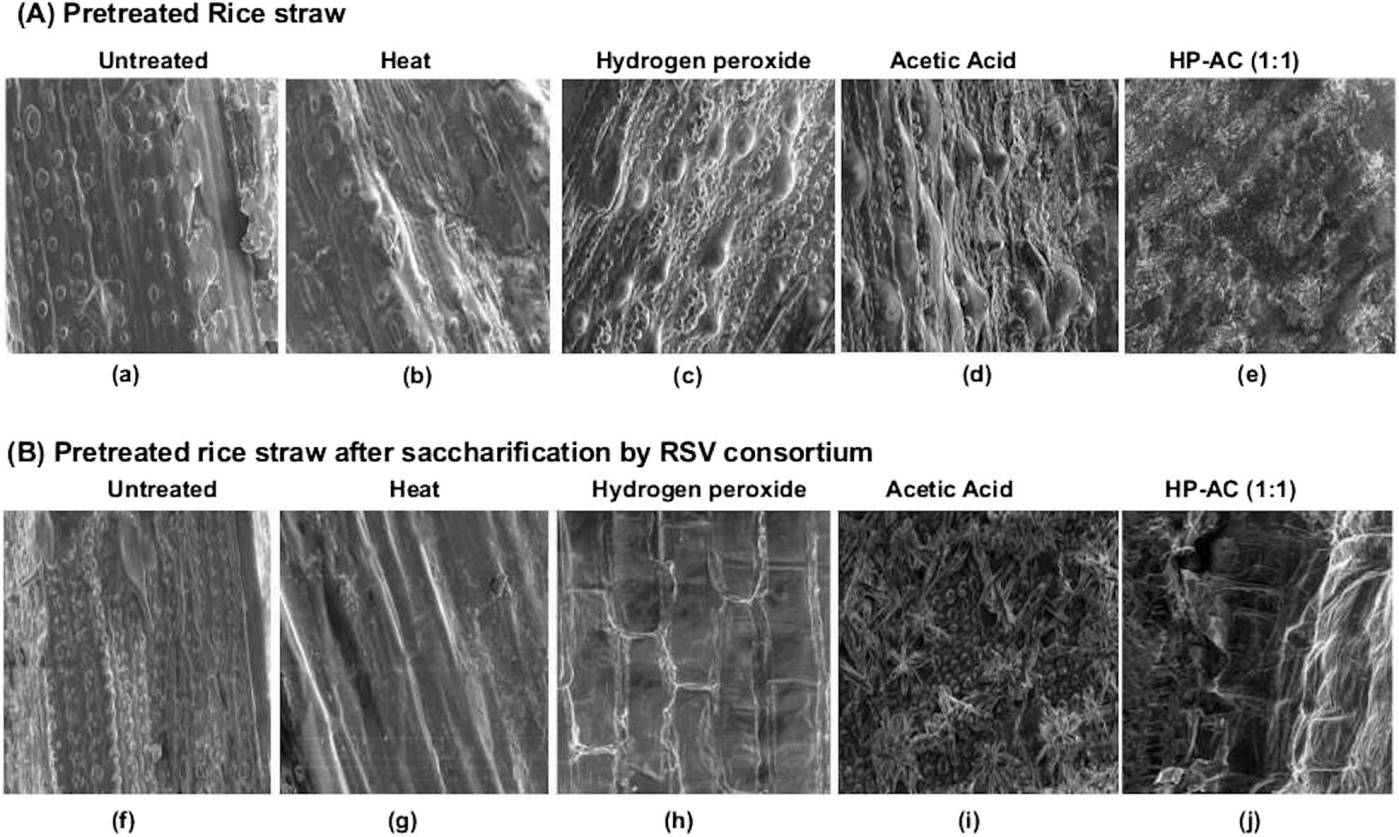
Scanning electron micrographs of pretreated rice straw before (Panel **A**, a–e) and after saccharification with RSV secretome (Panel **B**, f–j).

**Figure 4:**
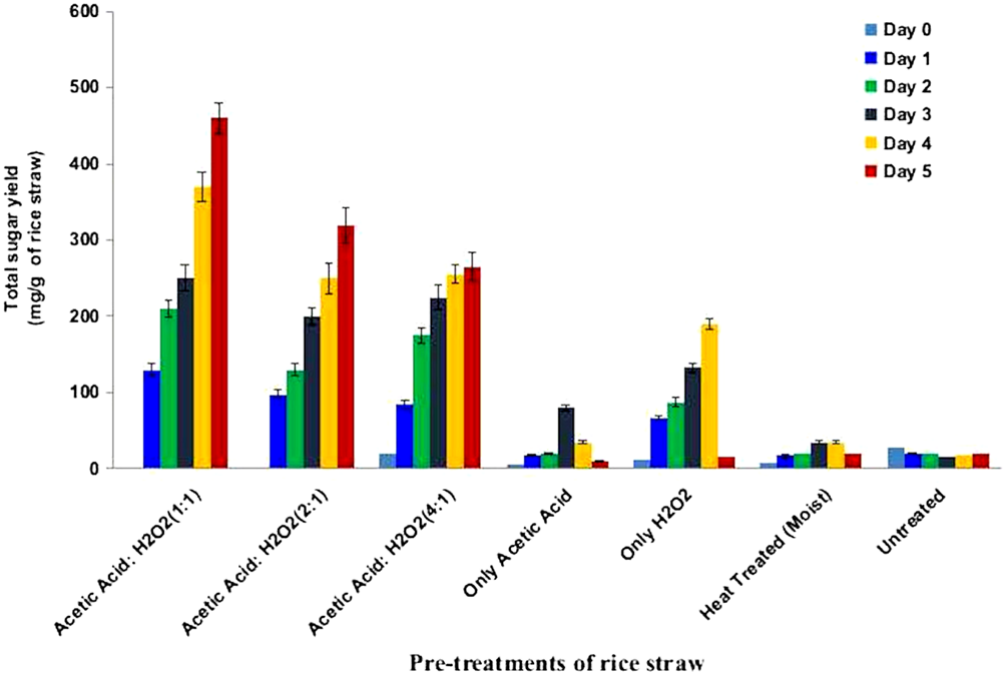
Saccharification of chemical and heat pretreated rice straw by RSV secretome.

The ability of RSV to saccharify cellulosic components of rice straw was further studied. For this, the processed rice straw was pretreated by various methods, like moist heat, hydrogen peroxide (HP), acetic acid (AC), and HP-AC containing different ratio of HP and AC; and the pretreated samples were used as substrates for saccharification by RSV secretome. Previous studies on organo-solvent pretreatment have shown that the HP-AC pre-treatment is more effective at increasing the enzymatic saccharification of lignocellulose biomass and therefore, various ratios of these two chemicals were used^13,18^. The morphological changes in pretreated rice straw samples before and after the RSV catalyzed saccharification was compared by scanning electron microscopy (SEM) (Fig. 3). The untreated rice straw showed a highly compact and rigid structure with abundance of silica nodules on the surface (Fig. 3a). Pretreatment with heat, HP and AC resulted in disruption of lignocellulose organization with incomplete/partial exposure of cellulose fibrils to the surface (Fig. 3b–d). The HP-AC (1:1) pretreatment led to effective delignification and loosening of rice straw with the formation of troughs and cracks on the surface (Fig. 3e) and was correlated with relatively greater deconstruction of cellulosic fibres on saccharification by RSV secretome (Fig. 3f–j). As observed in the present study, the HP-AC (1:1) pretreatment strategy has been shown to decrease the lignin content of rice straw by 85.12% and thus improving the downstream biocatalytic hydrolysis of cellulosic component with lower generation of fermentation inhibitors^13^. The results in Fig. 4 show that the RSV secretome catalyzed the release of reducing sugar from the rice straw. The amount of released glucose equivalents (RGE) was very low in case of either individually heat, HP and AC pretreated or untreated rice straw. On the other hand, pretreatment of processed rice straw having HP-AC, having HP and AC ratio of 1:1, 1:2 and 1:4 yielded RGE of 460, 320 and 260 mg g^−1^ of rice straw, respectively. The reduced RGE yield in case of rice straw pretreated with HP-AC with greater proportion of acetic acid (1:2 and 1:4) might be due to significantly lesser retention of hemicellulose by the pretreatment. As in the present study, Wi et al. reported the pretreatment strategy comprising 1:1 ratio of HP and AC with better yield of reducing sugars by the synergistic effect of the chemicals^13^. Moreover, a progressive increase in RGE yield upto 5 days of reaction at 60 °C indicated moderately thermotolerant nature of RSV secretome.

### Microbial diversity of the RSV consortium

The RSV consortium showing the ability to produce cellulolytic enzymes with saccharification potential was further analyzed for the microbial diversity, and CAZyme encoding genes associated with lignocellulose depolymerization. Metagenome sequencing of RSV in total generated 18, 831, 906 reads and their de novo assembly using MetaSPAdes program^19^ obtained 22,185 contigs, of which 15,601 contigs were atleast 200 bp in length and 10,308 contigs were more than 500 bp in length. The metagenome sequence has been deposited at DDBJ/ENA/GenBank under the accession number JAAROH010000001-JAAROH010015601. The RSV metagenome contained 41,930 open reading frames (ORFs) with average length of 763 bp. The microbial diversity of the RSV consortium was determined by identifying the comparative taxonomic abundance of all the contigs before removing the duplicates, using BLASTN^20^ search of the assembled contigs (> 200 bp) against the reference genome sequences in the NCBI-NR database with e-value cut off 10^–5^.

**Figure 5:**
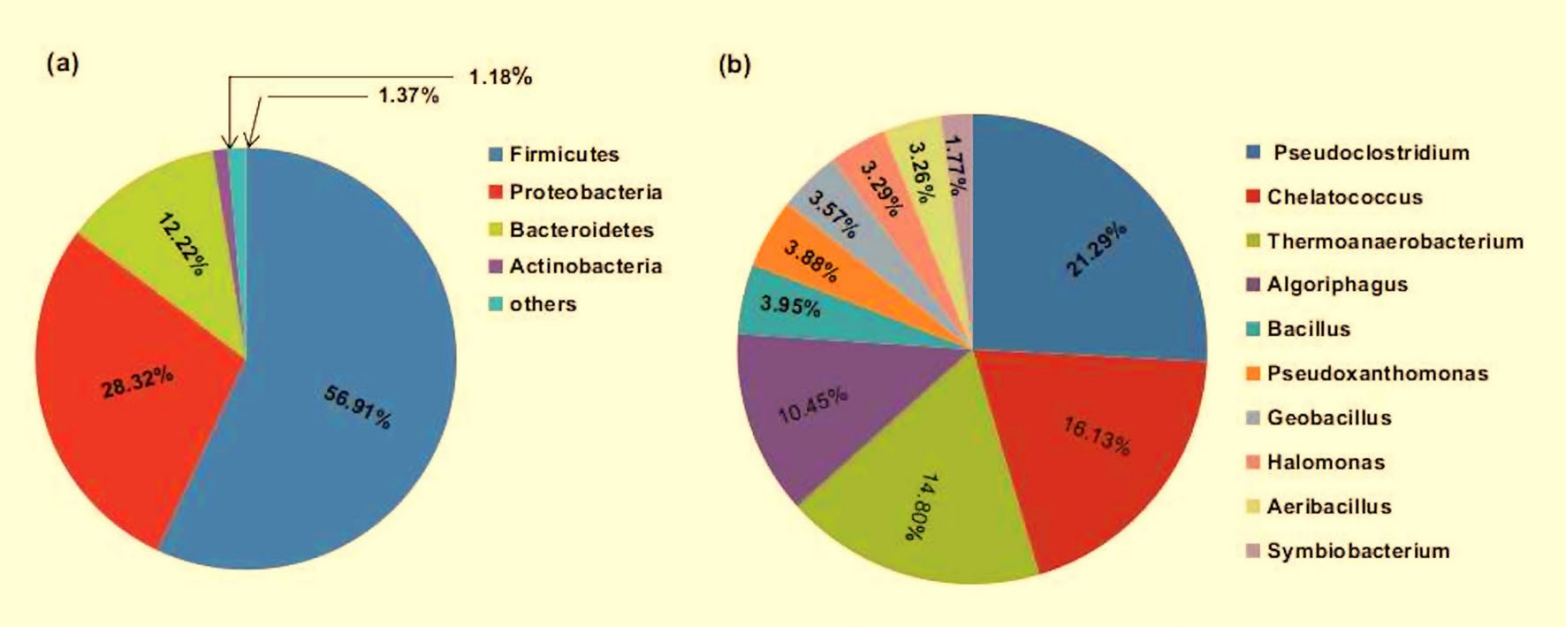
Taxonomic origin of bacteria in RSV consortium (**a**) Phylum and (**b**) Genus levels.

Based on NCBI attributes, the RSV consortium was found to be predominated by bacteria (83.82%) along with very few Archaea and Eukarya (1.18%); and rest 15% remained unclassified. The consortium was harboured by altogether 13,077 Eubacteria and 114 Archaebacteria. Among them the majority (56.91%) was assigned to phylum Firmicutes, followed by 28.32% to Proteobacteria and 12.22% to Bacteroidetes. Other bacteria belonging to phyla Actinobacteria, Euryarchaeota, Deinococcus-Thermus, Cyanobacteria, Spirochaetes, and Thermotogae showed least abundance (Fig. 5a, Additional File 1: Table S1). A comparison of the taxonomic profile of RSV with other publicly available metagenomes revealed that the microbial community of RSV consortium was similar to that of wallaby gut dominated by Firmicutes with high capacity to degrade plant cell wall^21^ and was quite different from other lignocellulolytic communities, like corn stover adapted compost with predominance of Proteobacteria^10^, and rice straw adapted compost with highest abundance of Actinobacteria^1^. Although composting and vermicomposting are organic waste decomposition processes comprising both aerobic and anoxic regions, the observed taxonomic disparity of RSV from other compost consortia might be due to the variation in the composting process of vermicompost as well as the adaptation strategy employed. The increased Firmicutes, Proteobacteria and Bacteroidetes populations could be derived from enrichment of the RSV with bacterial load of earthworm gut casting^9^ that survived at higher temperature. In earlier study the bacterial groups from the earthworm intestinal system were found to be mainly affiliated to the Proteobacteria, Firmicutes, Bacteroidetes, Actinobacteria, and Spirochetes^22^.

The RSV consortium showed the abundance of bacterial genera *Pseudoclostridium* (21.29%), *Chelatococcus* (16.13%), *Thermoanaerobacterium* (14.80%), *Algoriphagus* (10.45%), *Bacillus* (3.95%), *Pseudoxanthomonas* (3.88%), *Geobacillus* (3.57%), *Halomonas* (3.29%), *Aeribacillus* (3.26%) and *Symbiobacterium* (1.77%) (Fig. 5b, Additional file 2: Table S2). Previous studies reported the presence of most of these genera in thermal environments, like compost and hot spring^23,24^. The recently characterized genus *Pseudoclostridium* represented by the only species *Pseudoclostridium thermosuccinogenes,* is an anaerobic, thermophilic, xylanolytic bacteria belonging to the family *Hungateiclostridiaceae*. It is the only known thermophile producing succinic acid as one of the major products of fermentation^23^. Some *Chelatococcus* species are moderately thermophilic, aerobic, Alphaproteobacteria that generally grow abundantly in thermophilic lignocellulose degradation environment^23^. They can use succinate as carbon source and therefore, their presence in higher numbers in the consortium might correlate with the abundance of succinate producing *Pseudoclostridium*. Although *Chelatococcus* species are not reported to produce enzymes for cellulose hydrolysis, they trigger/activate the process through breakdown of recalcitrant lignin^25^. In earlier studies, *Thermoanaerobacterium* species have been reported as proficient degrader of plant biomass and they also show the capability to quickly convert crystalline cellulose to alcohols and volatile organic acids^26^. Several species of *Algoriphagus* were mostly isolated from saline and fresh water environments. They are heterotrophic, aerobe belonging to phylum Bacteroidetes and have shown the ability to degrade both the cellulose and hemicellulose polysaccharides^27^. *Pseudoxanthomonas* and *Geobacillus* species are reported to be present in varied habitat including lignocellulose degradation environments^28,29^. *Symbiobacterium* is a free living, cellulolytic, syntrophic genus which grows efficiently in co-culture with *Bacillus* species and its growth depends on high CO_2_ and low O_2_ conditions established by the precedence growth of the co-cultured bacteria^30^. The increased prevalence of *Symbiobacterium* might correlate with the predominance of *Bacillus* species in the RSV consortium. Although in our study the RSV was cultured under aerobic conditions, it contained both the aerobic and anaerobic bacteria. The results thus indicated bacterial synergism in the lignocellulose medium wherein aerobes and facultative anaerobes provided respiratory protection to cellulolytic anaerobes growing in co-culture and thus allowed synergistic utilization of cellulose even under an aerobic atmosphere.

### Functional profile of RSV metagenome

A preliminary knowledge of physiological functions of a complex microbial consortium can be obtained from the analysis of its metagenome for protein encoding ORFs. The RSV metagenome sequence was analyzed for functional abundance of protein encoding ORFs by using SEED^31^ and KEGG^32^ databases (Additional File 3: Fig S1a,b). The SEED annotation revealed that among the proteins with assigned functions, 14% were grouped in carbohydrate metabolism, 11% in nucleic acid metabolism, 9.65% in amino acid metabolism, 5.5% in energy metabolism and 3.65% in membrane transport. The functional analysis based on KEGG showed that the RSV consortium was enriched for carbohydrate metabolism (17%), amino acid metabolism (15.40%), DNA-RNA metabolism (11.63%), energy metabolism (8.44%) and membrane transport (6.35%). However, a large number of ORFs in the metagenome remained poorly characterized, with general or unknown functions. From the physiological function profile it is evident that the microorganisms of the RSV consortium on adapting to grow on rice straw, acquired improved ability of polysaccharide depolymerization, sugar transport and uptake. The results of KEGG based analysis of RSV metagenome was markedly different from that of RSA (rice straw adapted) metagenome derived from microbial community of rice straw adapted compost. For example, RSV had higher abundance carbohydrate metabolism genes (17%) than membrane transport genes (6.35%), whereas RSA showed almost equal abundance (12%) of genes of both the classes^1^.

### Abundance and diversity of CAZymes in RSV consortium

**Figure 6:**
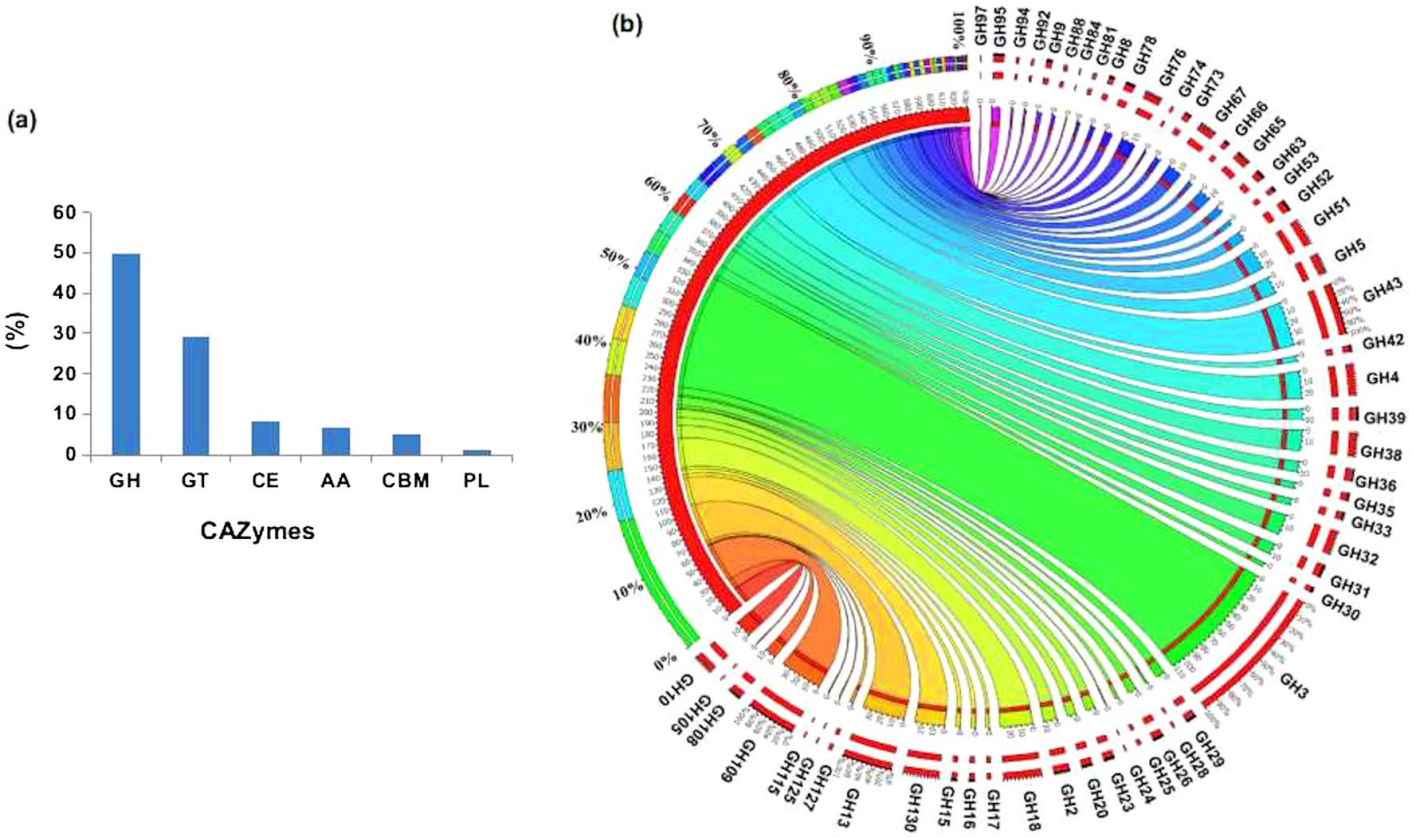
Distribution of lignocellulose depolymerizing CAZymes in RSV consortium. (**a**) Relative abundance of GHs, GTs, AAs, CBMs and PLs, and (**b**) Distribution of glycoside hydrolases (GH) as visualized by Circos software. The width of the bars from each GH family indicates its relative abundance in the RSV consortium.

Due to inherent complexity and heterogeneity, the complete depolymerization of lignocellulose biomass depends on the synergistic action of several CAZymes. Annotation of the RSV metagenome using the dbCAN database^33^ revealed the presence of 1389 CAZyme encoding ORFs with lignocellulolytic function. These ORFs were distributed between GH (49.63%), GT (29.32%), CE (8.28%), AA (6.48%) CBM (5.25%), and PL (1.13%) (Fig. 6a, Additional file 4: Table S3).

GH family enzymes play essential role in catalyzing the complete hydrolysis of cellulose and hemicellulose polymers to various types of monosaccharides that are utilized as energy sources by aerobic and anaerobic bacteria. The distribution of various GHs in RSV consortium as determined by Circos software^34^ has been shown in Fig. 6b. Although RSV metagenome contained 693 ORFs belonging to 55 different GH families, only 50% of them could be functionally characterized (Additional file 5: Table S4). The cellulolytic system consists of three major classes of hydrolytic enzymes; endoglucanase randomly acts on the internal sites in the cellulose chain; cellobiohydrolase or exoglucanase progressively acts on the reducing or non-reducing terminus of the cellulose chain and thereby releasing cellobiose and glucose; and β-glucosidase hydrolyzes cellobiose or cello-oligosaccharides to glucose^5,10,22^. The RSV metagenome contained 3 ORFs for GH8 and GH9 endoglucanases, 3 ORFs for GH1 and GH10 exoglucanases and 110 ORFs for β-glucosidases of GH1 (17), GH3 (76), GH4 (15) and GH94 (2) families. Although sixteen other cellulase family ORFs belonging GH5 family were also identified in the metagenome, they could not be assigned to specific cellulase types. The heterogeneous hemicellulose polysaccharide made of xylan, xyloglucans and mannans, is completely hydrolyzed by the combined action of enzymes with different substrate specificities, like endo-1,4-β-D-xylanase,1,4-β-D-xylosidase***, ***α-L-arabinofuranosidase, endo-1,4-α-mannosidase, acetyl xylan esterase and α-glucuronidase^10,22^. RSV metagenome identified several ORFs for enzymes associated with xylan depolymerization. These included 17 ORFs for GH8 (4) and GH10 (13) endoxylanases that cleave glycosidic linkages in the xylan backbone to release xylobiose and xylo-oligosaccharides. The activity of 1,4-β-D-xylosidase releases β-D-xylopyranosyl residues from the non-reducing terminus of xylobiose and small 4-β-D-xylooligosaccharides. The metagenome contained 8, 1 and 11 ORFs for1,4-β-D-xylosidases of families GH39, GH43 and GH52, respectively. A higher abundance of ORFs encoding xylan debranching enzymes were also mapped. The downstream removal of L-arabinose from arabinoxylan is catalyzed by α-L-arabinofuranosidase, whereas glucuronic acid residue from glucuronoxylan is hydrolyzed by α-glucuronidase. In the RSV metagenome all the detected α-L-arabinofuranosidases were encoded by GH51 (22 ORFs) family; and all the detected α-glucoronidases were encoded by GH67 (14 ORFs) family. The results of RSV metagenome analysis therefore revealed a greater abundance of ORFs for xylanases (26% of total GHs) than that for cellulases (13% of total GHs) which correlate with significantly greater in vitro activity of xylanases in comparison to cellulases in the present study.

Acetyl groups are common substituent in xylan and their removal is often key to effective endo-hemicellulase activity. Acetyl xylan esterase belonging to the CAZyme family CE cleaves O-acetyl groups from positions 2 and/or 3 on the β-D-xylopyranosyl residues of acetyl xylan and thus facilitates solubilization of the polymer^10,22^. The RSV metagenome sequence contained 12 acetyl xylan esterase encoding ORFs of CE7 family. In addition, presence of several other polysaccharide esterase ORFs belonging to families CE1, CE4 and CE10 indicated efficient degradation of xylan by the RSV consortium.

Lignin, a complex heteropolymer comprised of networks of aromatic phenylpropanoid units, imparts recalcitrance to cellulosic depolymerization. Auxiliary activity (AA) is a comparatively recently defined class of CAZyme containing various families of lignin, polysaccharide and oligosaccharide degrading redox enzymes. The criteria for integration of AAs within CAZyme happens to be their potential ability to help the GH and CE enzymes gain access to the complex cellulosic polymers of the plant cell wall^35^. The RSV consortium contained 80 ORFs with assigned AA functions and they were multicopper oxidase (AA1, 16.25%), catalase/peroxidase (AA2, 6.25%), GMC oxidoreductase (AA3, 50%), vanillyl alcohol oxidase (AA4, 10%), radical-copper oxidase (AA5, 6.25%) and quinone reductase (AA6, 11.25%); however, AA ORFs belonging to other families were absent.

### Phylogenetic affiliation of lignocellulolytic CAZymes in RSV consortium

**Figure 7:**
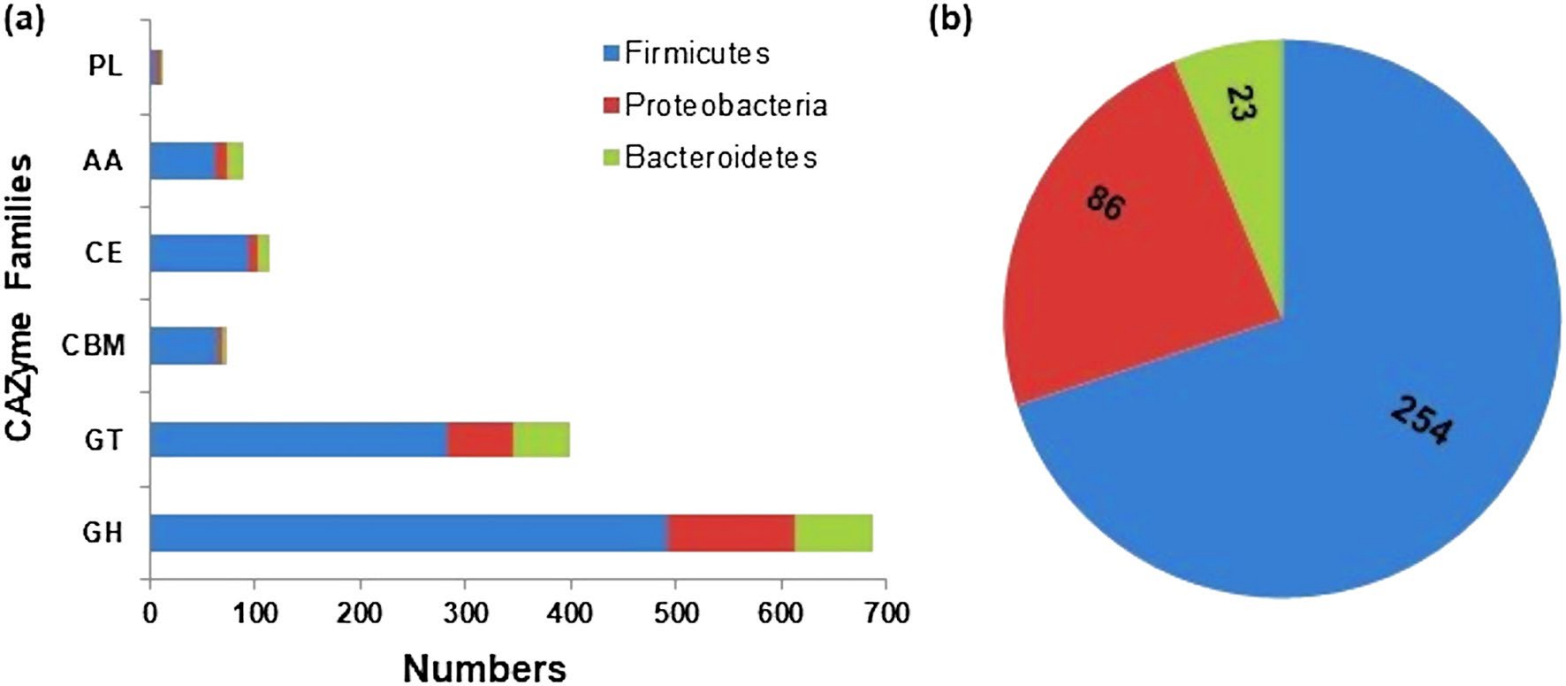
Phylogenetic distribution of CAZymes in RSV consortium. (**a**) Relative abundance of total CAZymes of GHs, GTs, CBMs, CEs and AAs families in various phyla and (**b**) Distribution of functionally assigned lignocellulolytic GHs, CEs and AAs in various phyla.

The phylogenetic distribution of CAZyme ORFs in the RSV metagenome was determined in order to understand the role of various microorganisms in lignocellulose decomposition process. Most of the CAZymes were derived from phyla Firmicutes, Proteobacteria and Bacteroidetes with the genera belonging to Firmicutes contributing markedly greater number of enzymes (Fig. 7a, Additional file 4: Table S3). The result is strikingly different from the previous research on the RSA consortium enriched from compost depicting the major role of cellulolytic enzymes produced by the members of phylum Actinobacteria in overall degradation process^1^. As in the present study, corn stover adapted microbial consortium enriched from compost harboured a broad array of cellulolytic ORFs in the representatives of Firmicutes despite having Proteobacteria as the predominant phylum^10^. Furthermore, the phylogenetic distribution of cellulolytic ORFs in the RSV metagenome was investigated at lower taxonomic level. Out of 484 bacterial genera identified in the consortium, only 72 genera encoded the CAZymes ORFs. Furthermore, the distribution of functionally characterized lignocellulolytic ORFs between various genera was determined (Additional file 5: Table S4). Altogether 363 lignocellulolytic ORFs with known GH, CE and AA functions were identified in various bacterial genera, of which 69.97% were encoded by representatives of Firmicutes, 23.70% by Proteobacteria, and 6.33% by Bacteroidetes (Fig. 7b). The microorganisms of phyla Firmicutes and Proteobacteria contained 43 and 57% of functionally assigned cellulolytic GHs encoding ORFs, respectively. On the other hand, the hemicellulolytic GH ORFs were mainly (95%) originated from the members of Firmicutes and the rest 5% were from Protobacteria and Bacteroidetes. The ORFs for the AA enzymes were more prevalent in the members of Firmicutes (63.10%) than that of Bateroidetes (23.80%) and Proteobacteria (13.10%).

**Figure 8:**
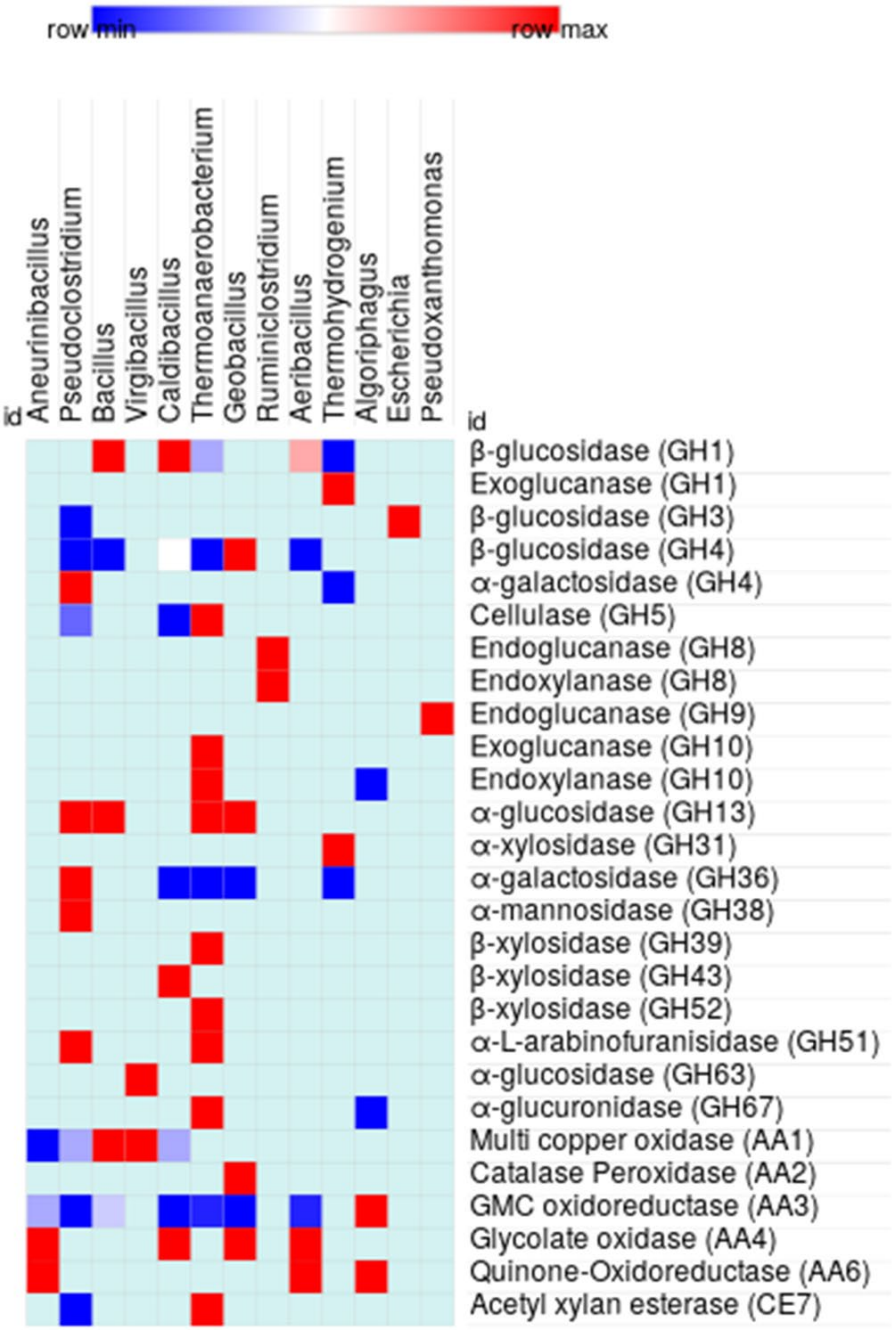
Heat map showing the distribution of glycoside hydrolase (GH) and auxillary activities (AA) families in bacterial genera in the RSV consortium. Only the GH families targeting cellulose, hemicelluloses and lignin are taken into account.

**Figure 9:**
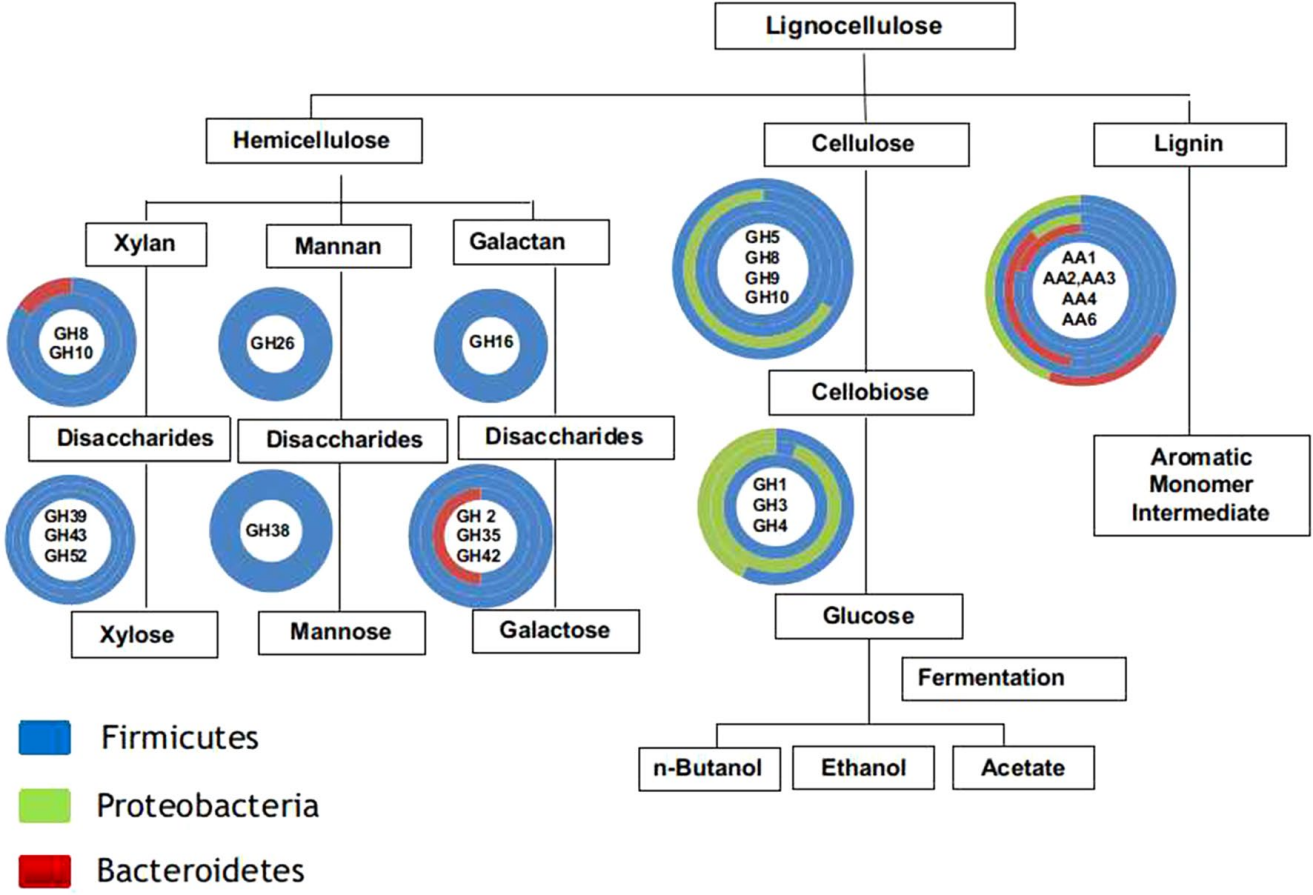
Lignocellulose degradation pathway and its related enzymes found in bacteria identified at the genus level in the RSV consortium. The relative abundance of GH families in representatives of bacterial phyla has been shown as concentric circles with inner circle corresponding to first GH in the list.

The results in Fig. 8 (Additional file 6: Table S5) show the abundance of functionally identified ORFs with lignocellulolytic activities in 13 bacterial genera of Firmicutes (10), Proteobacteria (2) and Bacteroidetes (1). Endoglucanases and exoglucannases belonging to GH1, 5 and 10 families were originated from members of Firmicutes like, *Thermoanaerobacterium* (13), *Pseudoclostridium* (3), *Caldibacillus* (1) and *Thermohydrogenium* (1), whereas the enzymes of GH8 and GH9 were encoded by Proteobacteria (*Pseudoxanthomonas*) and Firmicutes (*Ruminiclostridium*). The activity of β-glucosidase has been considered as a key factor in the cellulolytic process^1,22^. Several members of the RSV consortium encoded β-glucosidase of GH1, 3 and 4 families. The β-glucosidase ORFs of GH3 family were present in Proteobacteria (*Escherichia*) and Firmicutes (*Pseudoclostridium*), whereas phylogenetic distribution of GH1 and GH4 ORFs was more diverse and most of them originating from representatives of Firmicutes, like *Thermoanaerobacterium*, *Bacillus*, *Caldibacillus*, *Aeribacillus, Geobacillus* and *Thermohydrogenium*. *Thermoanaerobacterium,* one of the predominant member of RSV consortium (14.80%), possessed 89 functionally identified ORFs and among these 77.79, 17.97 and 2.24% were associated with hemicellulose, cellulose and lignin depolymerization, respectively. Similarly, *Pseudoclostridium* constituting 21.29% of RSV consortium encoded 57 functionally identified ORFs of which 77.19, 17.54 and 5.27% had hemicellulolytic, cellulolytic and lignolytic functions, respectively. The process of delignification catalyzed by AAs has been reported to be essential for efficient saccharification of plant lignocellulosic biomass^35^. The bacterial genera identified in the consortium altogether harboured 86 ORFs of AA1, AA2, AA3, AA4, AA5 and AA6 families. These AAs were mostly derived from aerobes or facultative anaerobes belonging to Firmicutes (65.52%), Proteobacteria (22.99%) and Bacteroidetes (11.49%). The Firmicutes, like *Aneurinibacillus*, *Bacillus*, *Aeribacillus*, *Caldibacillus* and *Geobacillus* contained ORFs for multiple families of AAs. *Algoriphagus*, a Bacteroidetes of high abundance (10.45%) in the RSV consortium, encoded 20% of the total functionally identified AAs. Although *Chelatococcus*, *Pseudoxanthomonas* and *Halomonas* from Protobacteria, and *Symbiobacterium* from Firmicutes, were the most abundant microorganisms in the RSV consortium and they encoded several CAZyme ORFs of hemicellulolytic and cellulolytic GH families, only few of them could be assigned with specific functions. Recently, *Chelatococcus* has been reported to have important role in delignification process^25^; however, only one AA encoding ORF from the genus could be identified. The results of the present study thus suggested the metabolic coordination between aerobic, facultative anaerobic and anaerobic microorganisms of RSV consortium in the decomposition of rice straw, which is schematically represented in Fig. 9. The depolymerization of hemicellulose was mainly carried out by anaerobic Firmicutes with very few contributions from representatives of Bacteroidetes and Proteobacteria. On the other hand, facultative anaerobic Proteobacteria, and aerobic and anaerobic Firmicutes were mainly associated with degradation of cellulose. The members of aerobic Firmicutes and Bacteroidetes together contributed to the process of delignification of rice straw.

## Conclusion

In the present research work, a thermotolerant microbial consortium (RSV) was developed from vermicompost for depolymerization of rice straw polysaccharides. RSV produced various cellulases and xylanases with higher activity at 60 °C. Furthermore, enzymes present in RSV secretome catalysed saccharification of 1:1, H_2_O_2_:CH_3_COOH pretreated rice straw. The RSV metagenome analysis revealed the presence of several cellulolytic and non-cellulolytic microorganisms. The members of Firmicutes, Proteobacteria and Bacteroidetes were in greater abundance and they harboured several CAZyme ORFs. The Firmicutes had major role in the degradation of hemicellulose, cellulose and lignin, whereas representatives of Proteobacteria, and Bacteroidetes mainly contributed to the depolymerization of cellulose and lignin, respectively. The results thus suggested metabolic coordination between cellulolytic CAZymes of various phyla in rice straw deconstruction. Hence, the RSV consortium could be a resource for mining thermotolerant cellulolytic bacteria and/or enzymes and studying their synergism in deconstruction of chemically pretreated rice straw.

## Methods

### Development of rice straw adapted microbial consortium (RSV) from vermicompost

The compost inoculum was collected from the green waste vermicomposting facility at the Centre for Floriculture and Agri-business Management, University of North Bengal. Rice straw purchased from the local market was thoroughly washed, air dried, cut into small (6 ± 2 mm) pieces and stored in zipper lock bags at 4 °C until use. Vermicompost (1% w/v) was inoculated into M9-rice straw medium containing in g l^−1^, Na_2_HPO_4,_ 6; KH_2_PO_4_, 3; NaCl, 5; NH_4_Cl, 1; MgSO_4_, 0.50; CaCl_2_,0.015 and 3% (w/v) processed rice straw as carbon source, pH 6.5 and grown at 37 °C under 150 rpm shaking for one week. The culture was adapted to grow at higher temperature by sub-culturing (1% v/v) weekly into fresh M9 rice straw medium at gradually increasing temperature. For this the culture was sub-cultured weekly at 45 °C for two weeks and then subcultured weekly at 55 °C for 2 weeks. Following this, it was sub-cultured for ten weeks at 60 °C. The tenth passage of the culture grown at 60 °C, designated as RSV, was further used for various experiments.

### Agarose well diffusion assay of cellulase and xylanase

RSV culture was centrifuged at 11,000×*g* for 15 min and the clarified cell free supernatant (CFS) obtained after passing through 0.2 µm filter was used as the source of extracellular enzymes for Agarose well diffusion assay^36^. 1% agarose gel supplemented with either 0.5% carboxy methyl cellulose (CM-cellulose) or 0.5% birchwood xylan, was cast on glass plate and wells (7 mm diameter) were made. After adding 70 μl of CFS (3 μg extracellular protein) to each well, the xylan plate was incubated at 60 °C for 30 min and CM-cellulose plate was incubated at 60 °C for 24 h. The gels were stained with 0.1% congo-red for 15 min followed by destaining with 1 M NaCl for another 15 min. Then the gels were visualized for presence of hydrolytic zones around the wells.

### Enzyme assays and protein estimation

The CFS was obtained from the RSV culture at 24 h intervals for four consecutive days of growth and assayed for xylanases (endoxylanase, β-xylosidase, α-L-arabinofuranosidase) and cellulases (cellobiohydrolase, endoglucanase, and β-glucosidase) in the pH range 5–8 and at reaction temperatures 37 and 60 °C. All the assays were carried out in the following buffers: 100 mM citrate buffer (pH 5 and 6) and 100 mM phosphate buffer (pH 7) and 100 mM Tris–HCl (pH 8). Endoxylanase and endoglucanase activities were measured by the reducing sugar method using their respective substrates, 1% birchwood xylan and 1% CM-cellulose. The reducing sugar was estimated by using 3,5-dinitrosalicylic acid (DNS) reagent^37^. The assay mixture in total volume of 1 ml contained 1% substrate, 80 mM buffer and 100 μl CFS. Endoxylanase reaction was incubated for 30 min, whereas endoglucanase reaction was incubated for 24 h. Both the reactions were stopped by the addition of 1 ml DNS reagent followed by incubation in a boiling water bath for 10 min. The amount of reducing sugar released was determined by measuring absorbance at 540 nm. One unit (U) of enzymatic activity refers to the amount of enzyme that released 1 μmol of xylose or glucose 30 min^−1^, under standard assay conditions.

The activities of β-glucosidase, β-xylosidase, cellobiohydrolase and α-L-arabinofuranosidase were assayed by using their respective substrates, p-nitrophenyl-β-D-glucopyranoside, p-nitrophenyl-β-D-xylopyranoside, p-nitrophenyl-β-D-cellobioside and p-nitrophenyl-α-L-arabinofuranoside. The reaction mixture contained 2.5 mM of substrate, 80 mM buffer, and 100 μl CFS. After incubation at specified temperatures for 24 h, the reaction was terminated by adding 600 μl of 0.4 M NaOH-glycine buffer (pH 10.8) and the amount of p-nitrophenol (pNP) released was measured at 405 nm using pNP as reference. The reaction mixture without CFS incubated for 24 h at specified temperatures served as control. One unit of enzyme activity refers to the amount of enzyme that released 1 μmol of pNP 30 min^-1^ under standard assay conditions. Protein concentration was determined by the dye binding method of Bradford using bovine serum albumin as standard^38^. All the enzymatic assays and protein estimation were carried out using three biological replicates.

### Pretreatment of rice straw and saccharification by RSV secretome

The RSV consortium was tested for its ability to saccharify moist–heat and chemically pretreated rice straw. For heat pretreatment, the processed rice straw was autoclaved at 121 °C and 15 psi for 30 min. The chemical pretreatment was done following the method of Wi et al.^13^ with some modifications. The processed rice straw (5% w/v) was suspended in either acetic acid (AA) or hydrogen peroxide (HP) or mixture of these two reagents in ratios of 1:1, 2:1, and 4:1 and then incubated at 60 °C for 6 h. Thoroughly washed pretreated rice straw samples were used as the substrate for saccharification. The RSV culture (tenth passage, day 2 incubation temperature 60 °C) was centrifuged at 11,000×*g* for 15 min and the CFS was used for preparation of secretome. To the CFS twice volume of acetone was added and incubated at − 20 °C for 4 h. The mixture was centrifuged at 11,000×*g* for 15 min. The resulting protein pellet was air dried, suspended in 100 mM citrate buffer (pH 6) and used as the enzyme preparation for saccharification of heat and chemical pretreated rice straw. The saccharification reaction in total volume of 10 ml contained 0.5 g pretreated rice straw, 9 ml of 100 mM citrate buffer (pH 6) and 1 ml (Cellulase-50 U ml^−1^, Xylanase-50 U ml^−1^) of enzyme preparation and a separate reaction was set for each sampling. The reaction was incubated at 60 °C and at specified time intervals the saccharification reaction was centrifuged at high speed and the amount of  released glucose equivalents (RGE) in the total volume of supernatent was determined using DNS reagent and a glucose standard curve. The reaction mixture without enzyme preparation served as control in order to estimate non-enzymatic degradation of the agro-residues at 60 °C. All saccharification experiments were conducted using three biological replicates and RGE mean values and standard deviations are presented.

### Scanning electron microscopy (SEM) analysis

The rice straw samples after pretreatment and after subsequent saccharification by the RSV secretome were fixed with glutaraldehyde, dehydrated with increasing alcohol concentrations and mounted on stainless steel round stub using carbon tape. Then the samples were sputter coated with gold (Au) nanoparticles in an ion-sputter coater before viewing under SEM at 10 mm and 5 kV acceleration voltage and 1000 × magnification.

### Isolation of genomic DNA

Genomic DNA was extracted from the tenth passage (day 2) RSV culture by the method of Verma and Satyanarayan^39^. The culture filtrate obtained after passing through Mira-cloth (Merck) was centrifuged at 11,000xg for 10 min. One gram cell pellet was suspended in 2 ml of extraction buffer [*N*,*N*,*N*,*N*-cetyltrimethylammonium bromide (CTAB), 1%; polyvinylpolypyrrolidone (PVP), 2%; NaCl, 1.5 M; EDTA,100 mM; TE buffer pH 8.0, 0.1 M; sodium phosphate buffer (pH 8.0), 0.1 M; RNase A (10 mg ml^−1^), 100 μl and proteinase K (10 mg ml ^- 1^), 20 μl] and the suspension was incubated at 37 °C for 15 min at 200 rpm shaking; and then 200 µl of 10% SDS was added. The lysate was incubated at 60 °C for 1 h with intermittent shaking and then centrifuged at 11,000×*g* for 10 min. DNA was precipitated from the supernatant by adding 1 ml PEG (30% in 1.6 M NaCl), keeping at room temperature (RT) for 1 h and then centrifuging at 11,000×*g* for 10 min. DNA pellet was washed with 75% ethanol, dried at RT and dissolved in 100 µl of sterile Milli Q water. The extracted DNA quantity was 70 µg and had 260/280 ratio of 1.60.

### Metagenome sequencing

DNA library of 250 bp size was constructed using Nextera DNA Library preparation kit (Illumina, USA) and sequenced on Illumina HiSeq 2500 platform. Sequence reads were preprocessed using FASTX-toolkit^40^ for base and sequence quality score distributions, average base content per read and GC distribution; and duplicate reads were identified and removed. Adapter trimming was performed using Cutadapt (version 1.8.1)^41^. The de novo assembly of the quality filtered raw reads was carried out using MetaSPAdes (version 3.10.1)^19^ with default k-mer sizes of 21, 33 and 55.

### Functional annotation of metagenome database

The assembled contigs of atleast 200 bp size were used as input to Meta Gene Annotator (MGA)^42^ to predict ORFs; and to distinguish them from noncoding DNA. Functional annotation of ORFs was performed by DIAMOND BLASTX search^43^ against NCBI-NR database, with optimum e-value of 1e^−5^. Further, functional annotation of the predicted ORFs was carried out by BLASTP search against the KEGG^31^ and SEED^32^ database with e-value of 1e^−5^.

### Taxonomic profiling of metagenome database

The metagenome sequence of the RSV consortia was taxonomically profiled at genus level by using BLASTN^20^ search of the assembled contigs against NCBI non-redundant taxonomy databases of reference sequences of bacteria, archaea, eukaryote, viruses, unclassified and other sequences with e-value cut off 10^–5^. The hierarchy of comparative taxonomic abundance of various genera was determined at various taxonomic levels.

### Carbohydrate active enzymes (CAZymes): annotation and phylogenetic analysis

Carbohydrate-active enzymes (CAZymes) encoding genes in the metagenome (E < 10^–4^) were annotated using dbCAN (http://csbl.bmb.uga.edu/dbCAN/) based on the hidden markov model (HMMs) of the signature domain of each CAZy family^33^. The predicted CAZymes in the metagenome were further searched against the NCBI non-redundant database via BLASTX for functional annotation of lignocellulolytic activities and their phylogenetic affiliation. The functionally assigned lignocellulolytic ORFs were further analysed by Circos^34^ and Clustvis^44^.

## Supplementary Information

Supplementary Legends.

Supplementary Table S1.

Supplementary Table S2.

Supplementary Fig. S1.

Supplementary Table S3.

Supplementary Table S4.

Supplementary Table S5.

## Data Availability

All data generated or analysed during this study are included in this published article (and its supplementary information files).
