# Supplementary material for: Functional characterization of thermotolerant microbial consortium for lignocellulolytic enzymes with central role of Firmicutes in rice straw depolymerization
Source: Sci Rep. 2021 Feb 4;11:3032. doi: 10.1038/s41598-021-82163-x (PMC7862241; doi:10.1038/s41598-021-82163-x)
Supplement: Supplementary file 1 — Supplementary Legends. [file 41598_2021_82163_MOESM1_ESM.docx]

**Supplementary Information**

**Additional file1: Table S1.** Relative abundance of bacterial phyla, classes and families in RSV consortium

**Additional file 2: Table S2.** Relative abundance of bacterial genera in RSV consortium

**Additional file 3: Fig S1.** Distribution profile of (a) KEGG and (b) SEED assigned proteins in the RSV consortium

**Additional file 4: Table S3.** CAZyme family enzyme ORFs identified in the metagenome of RSV consortium

**Additional file 5: Table S4.** Distribution of functionally identified ORFs of lignocellulolytic GHs, CEs and AAs activities in various bacterial genera

**Additional file 6: Table S5.** Base file for heat map showing the distribution of glycoside hydrolase (GH) and auxillary activities (AA) families in thirteen bacterial genera of the RSV consortium. Only the GH families targeting cellulose, hemicelluloses and lignin are taken into account.
