# Supplementary material for: Functional characterization of thermotolerant microbial consortium for lignocellulolytic enzymes with central role of Firmicutes in rice straw depolymerization
Source: Sci Rep. 2021 Feb 4;11:3032. doi: 10.1038/s41598-021-82163-x (PMC7862241; doi:10.1038/s41598-021-82163-x)
Supplement: Supplementary file 2 — Supplementary Table S1. [file 41598_2021_82163_MOESM2_ESM.pdf]

**Title:** Functional characterization of thermotolerant microbial consortium for lignocellulolytic enzymes with central role of firmicutes in rice straw depolymerisation

**Authors**

*Parmeshwar V. Gavande, E.mail: parmesh.gavande9@gmail.com

*Arijita Basak, E. mail: basakarijita.biotech@gmail.com

Subhajit Sen, E.mail: subhajit252@gmail.com

Khusboo Lepcha, E.mail: khusboo.microbiology@gmail.com

Nensina Murmu, E.mail: nensinamur75@gmail.com

Vijeta Rai, E. mail: vijetaarpan@gmail.com

Deepika Mazumdar, E.mail: deepikamazumdar@gmail.com

Shyama Prasad Saha, E. mail: shyamaprasad.saha3@gmail.com

Vaskar Das, E.mail: dasvaskar303@gmail.com

[@]Shilpi Ghosh, E.mail: ghosshilpi@gmail.com; shilpighosh@nbu.ac.in

**Affiliation**: Department of Biotechnology, University of North Bengal, Raja Rammohunpur, P.O.-NBU, Siliguri, West Bengal, India, PIN-734013

*Contributed equally

[@]**Correspondence:** Department of Biotechnology, University of North Bengal, Raja Rammohunpur, Siliguri, West Bengal, India, PIN-734013

E. mail: ghosshilpi@gmail.com

shilpighosh@nbu.ac.in

**Phylum**

| | |
|---|---|
| Firmicutes | 7508 |
| Proteobacteria | 3736 |
| Bacteroidetes | 1613 |
| Actinobacteria | 156 |
| Euryarchaeota | 114 |
| Deinococcus-Thermus | 23 |
| Cyanobacteria | 14 |
| Chloroflexi | 7 |
| Spirochaetes | 5 |
| Thermotogae | 4 |
| Chlorobi | 2 |
| Fusobacteria | 2 |
| Verrucomicrobia | 2 |
| Acidobacteria | 1 |
| Gemmatimonadetes | 1 |
| Nitrospirae | 1 |
| Planctomyecetes | 1 |
| Tenericutes | 1 |
| | |
| Total phylum | 19 |

## class

| | |
|---|---|
| Clostridia | 5348 |
| Alphaproteobacteria | 2368 |
| Bacilli | 2148 |
| Sphingobacteriia | 1406 |
| Gammaproteobacteria | 1246 |
| Flavobacteria | 150 |
| Actinobacteria | 141 |
| Halobacteria | 112 |
| Betaproteobacteria | 106 |
| Cytophagia | 34 |
| Deinococci | 23 |
| Cyanophyceae | 14 |
| actinomycetales | 12 |
| Chitinophagia | 11 |
| Deltaproteobacteria | 10 |
| Bacteroidia | 8 |
| Dehalococcoidetes | 7 |
| Tissierellia | 6 |
| Spirochaetia | 5 |
| Diplococci | 4 |
| Thermotogae | 4 |
| Epsilonproteobacteria | 3 |
| Hydrogenophilalia | 3 |
| Bacteroidetes | 2 |
| Chlorobea | 2 |
| Fusobacteria | 2 |
| Negativicutes | 2 |
| Acidobacteria | 1 |
| Gemmatimonadetes | 1 |
| Methanomicrobia | 1 |
| Mollicutes | 1 |
| Nitriliruptoria | 1 |
| Nitrospira | 1 |
| Planctomycetacia | 1 |
| Rubrobacteria | 1 |
| Saprospiria | 1 |
| Thermococci | 1 |
| Thermoleophilia | 1 |
| Verrucomicrobiae | 1 |
| | |
| Total class | 40 |

**Family**

| | |
|---|---|
| Hungateiclostridiaceae | 2893 |
| Beijerinckiaceae | 2131 |
| Bacillaceae | 1862 |
| Cyclobacteriaceae | 1394 |
| Xanthomonadaceae | 528 |
| Halomonadaceae | 438 |
| Paenibacillaceae | 239 |
| Enterobacteriaceae | 143 |
| Cytophagaceae | 104 |
| Halobacteriaceae | 103 |
| Clostridiaceae | 95 |
| Vibrionaceae | 70 |
| Micrococcaceae | 65 |
| Flavobacteriaceae | 60 |
| Methylobacteriaceae | 59 |
| Tissierellaceae | 53 |
| Bradyrhizobiaceae | 43 |
| Thermoanaerobacteraceae | 40 |
| Rhodobacteraceae | 30 |
| Burkholderiaceae | 29 |
| Comamonadaceae | 27 |
| Alcaligenaceae | 25 |
| Deinococcaceae | 22 |
| Phyllobacteriaceae | 22 |
| Peptococcaceae | 19 |
| Corynebacteriaceae | 18 |
| Lachnospiraceae | 18 |
| Rhizobiaceae | 18 |
| Chitinophagaceae | 16 |
| Pseudomonadaceae | 15 |
| Ruminococcaceae | 15 |
| Sporolactobacillaceae | 14 |
| Actinomycetaceae | 12 |
| Rhodospirillaceae | 12 |
| Sphingobacteriaceae | 10 |
| Sphingomonadaceae | 10 |
| Acetobacteraceae | 9 |
| Dermabacteraceae | 9 |
| Methylococcaceae | 9 |
| Microbacteriaceae | 9 |
| Ectothiorhodospiraceae | 8 |
| Oxalobacteraceae | 8 |
| Planococcaceae | 8 |
| Thermoactinomycetaceae | 7 |
| Xanthobacteraceae | 7 |
| Enterococcaceae | 6 |

| | |
|---|---|
| Flammeovirgaceae | 6 |
| Listeriaceae | 6 |
| Aurantimonadaceae | 5 |
| Bacteroidaceae | 5 |
| Cryomorphaceae | 5 |
| Eubacteriaceae | 5 |
| Halorubraceae | 5 |
| Mycobacteriaceae | 5 |
| Nocardiaceae | 5 |
| Propionibacterineae | 5 |
| Rhodanobacteraceae | 5 |
| Rhodocyclaceae | 5 |
| Staphylococcaceae | 5 |
| Brucellaceae | 4 |
| Haloferacaceae | 4 |
| Moraxellaceae | 4 |
| Peptostreptococcaceae | 4 |
| Rhodobiaceae | 4 |
| Spirochaetaceae | 4 |
| Thiobacillaceae | 4 |
| Aeromonadaceae | 3 |
| Defluvitaleaceae | 3 |
| Geobacteraceae | 3 |
| Hydrogenophilaceae | 3 |
| Hyphomicrobiaceae | 3 |
| Neisseriaceae | 3 |
| Pasteurellaceae | 3 |
| Pseudonocardiaceae | 3 |
| Streptosporangiaceae | 3 |
| Syntrophomonadaceae | 3 |
| Thermotogaceae | 3 |
| Zoogloeaceae | 3 |
| Aphanothecaceae | 2 |
| Azotobacteraceae | 2 |
| Bernardetiaceae | 2 |
| Bifidobacteriaceae | 2 |
| Brevibacteriaceae | 2 |
| Campylobacteraceae | 2 |
| Caulobacteraceae | 2 |
| Chlorobiaceae | 2 |
| Chromatiaceae | 2 |
| Chromobacteriaceae | 2 |
| Coriobacteriaceae | 2 |
| Erwiniaceae | 2 |
| Fusobacteriaceae | 2 |
| Gottschalkiaceae | 2 |
| Lactobacillaceae | 2 |

| | |
|---|---|
| Methylocystaceae | 2 |
| Myxococcaceae | 2 |
| Nostocaceae | 2 |
| Peptoniphilaceae | 2 |
| Sporichthyaceae | 2 |
| Stigonemataceae | 2 |
| Streptococcaceae | 2 |
| Streptomycetaceae | 2 |
| Thioalkalispiraceae | 2 |
| Acidobacteriaceae | 1 |
| Actinopolysporaceae | 1 |
| Alcanivoracaceae | 1 |
| Alicyclobacillaceae | 1 |
| Baekduiaceae | 1 |
| Brachyspiraceae | 1 |
| Caldicoprobacteraceae | 1 |
| Cellvibrionaceae | 1 |
| Colwelliaceae | 1 |
| Cystobacteraceae | 1 |
| Dermatophilaceae | 1 |
| Dermocarpellaceae | 1 |
| Desulfobulbaceae | 1 |
| Egicoccaceae | 1 |
| Erythrobacteraceae | 1 |
| Ferrimonadaceae | 1 |
| Flexibacteraceae | 1 |
| Fortieaceae | 1 |
| Francisellaceae | 1 |
| Frankiaceae | 1 |
| Gemmatimonadaceae | 1 |
| Haliscomenobacteraceae | 1 |
| Haloanaerobiaceae | 1 |
| Heliobacteriaceae | 1 |
| Intrasporangiaceae | 1 |
| Legionellaceae | 1 |
| Lentimicrobiaceae | 1 |
| Marinifilaceae | 1 |
| Marinilabiliaceae | 1 |
| Methylacidiphilaceae | 1 |
| Micrococcineae | 1 |
| Microcystaceae | 1 |
| Micromonosporaceae | 1 |
| Microscillaceae | 1 |
| Morganellaceae | 1 |
| Muribaculaceae | 1 |
| Mycoplasmataceae | 1 |
| Nitrospiraceae | 1 |

| | |
|---|---|
| Opitutaceae | 1 |
| Ornithinimicrobiaceae | 1 |
| Oscillatoriaceae | 1 |
| Planctomycetaceae | 1 |
| Polyangiaceae | 1 |
| Prevotellaceae | 1 |
| Prochloraceae | 1 |
| Promicromonosporaceae | 1 |
| Pseudoalteromonadaceae | 1 |
| Rhodothermaceae | 1 |
| Rivulariaceae | 1 |
| Sandaracinaceae | 1 |
| Saprospiraceae | 1 |
| Schleiferiaceae | 1 |
| Scytonemataceae | 1 |
| Selenomonadaceae | 1 |
| Shewanellaceae | 1 |
| Sinobacteraceae | 1 |
| Solirubrobacteraceae | 1 |
| Sporomusaceae | 1 |
| Synechococcaceae | 1 |
| Thermaceae | 1 |
| Thermococcaceae | 1 |
| Thermomonosporaceae | 1 |
| Thiotrichaceae | 1 |
| Yersiniaceae | 1 |
| | |
| total family | 10975 |
