# Supplementary material for: Functional characterization of thermotolerant microbial consortium for lignocellulolytic enzymes with central role of Firmicutes in rice straw depolymerization
Source: Sci Rep. 2021 Feb 4;11:3032. doi: 10.1038/s41598-021-82163-x (PMC7862241; doi:10.1038/s41598-021-82163-x)
Supplement: Supplementary file 3 — Supplementary Table S2. [file 41598_2021_82163_MOESM3_ESM.pdf]

Title: Functional characterization of thermotolerant microbial consortium for lignocellulolytic enzymes with central role of firmicutes in rice straw depolymerisation

Authors

*Parmeshwar V. Gavande, E.mail: parmesh.gavande9@gmail.com

*Arijita Basak, E. mail: basakarijita.biotech@gmail.com

Subhajit Sen, E.mail: subhajit252@gmail.com

Khusboo Lepcha, E.mail: khusboo.microbiology@gmail.com

Nensina Murmu, E.mail: nensinamur75@gmail.com

Vijeta Rai, E. mail: vijetaarpan@gmail.com

Deepika Mazumdar, E.mail: deepikamazumdar@gmail.com

Shyama Prasad Saha, E. mail: shyamaprasad.saha3@gmail.com

Vaskar Das, E.mail: dasvaskar303@gmail.com

[@]Shilpi Ghosh, E.mail: ghosshilpi@gmail.com; shilpighosh@nbu.ac.in

Affiliation: Department of Biotechnology, University of North Bengal, Raja Rammohunpur, P.O.-NBU, Siliguri, West Bengal, India, PIN-734013

*Contributed equally

[@]**Correspondence:** Department of Biotechnology, University of North Bengal, Raja Rammohunpur, Siliguri, West Bengal, India, PIN-734013

E. mail: ghosshilpi@gmail.com

shilpighosh@nbu.ac.in

(a)

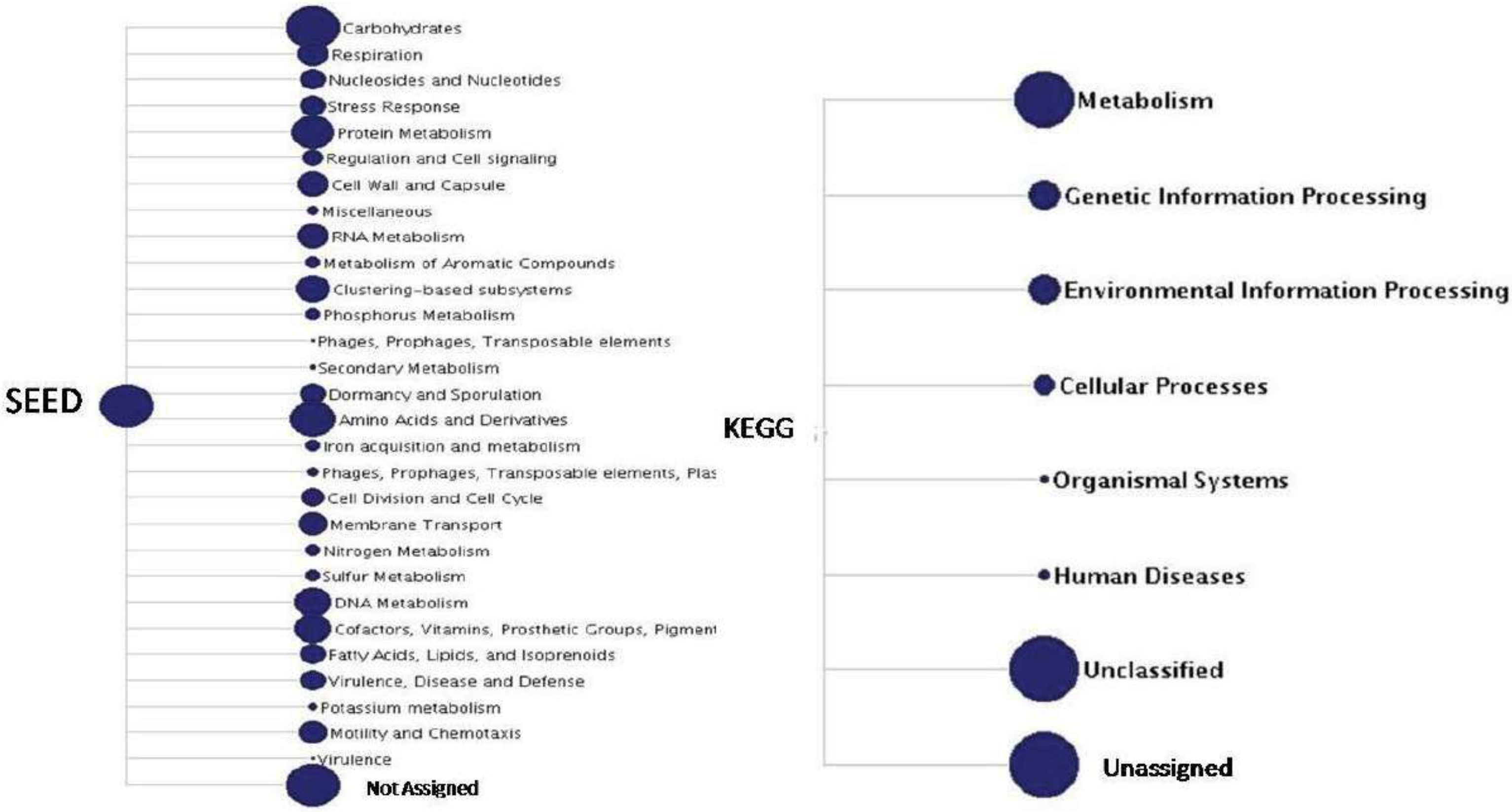

SEED
Carbohydrates
Respiration
Nucleosides and Nucleotides
Stress Response
Protein Metabolism
Regulation and Cell signaling
Cell Wall and Capsule
Miscellaneous
RNA Metabolism
Metabolism of Aromatic Compounds
Clustering-based subsystems
Phosphorus Metabolism
Phages, Prophages, Transposable elements
Secondary Metabolism
Dormancy and Sporulation
Amino Acids and Derivatives
Iron acquisition and metabolism
Phages, Prophages, Transposable elements, Plas
Cell Division and Cell Cycle
Membrane Transport
Nitrogen Metabolism
Sulfur Metabolism
DNA Metabolism
Cofactors, Vitamins, Prosthetic Groups, Pigment
Fatty Acids, Lipids, and Isoprenoids
Virulence, Disease and Defense
Potassium metabolism
Motility and Chemotaxis
Virulence
Not Assigned
KEGG
Metabolism
Genetic Information Processing
Environmental Information Processing
Cellular Processes
Organismal Systems
Human Diseases
Unclassified
Unassigned
