# Supplementary material for: Functional characterization of thermotolerant microbial consortium for lignocellulolytic enzymes with central role of Firmicutes in rice straw depolymerization
Source: Sci Rep. 2021 Feb 4;11:3032. doi: 10.1038/s41598-021-82163-x (PMC7862241; doi:10.1038/s41598-021-82163-x)
Supplement: Supplementary file 4 — Supplementary Fig. S1. [file 41598_2021_82163_MOESM4_ESM.pdf]

Title: Functional characterization of thermotolerant microbial consortium for lignocellulolytic enzymes with central role of firmicutes in rice straw depolymerisation

Authors

*Parmeshwar V. Gavande, E.mail: parmesh.gavande9@gmail.com

*Arijita Basak, E. mail: basakarijita.biotech@gmail.com

Subhajit Sen, E.mail: subhajit252@gmail.com

Khusboo Lepcha, E.mail: khusboo.microbiology@gmail.com

Nensina Murmu, E.mail: nensinamur75@gmail.com

Vijeta Rai, E. mail: vijetaarpan@gmail.com

Deepika Mazumdar, E.mail: deepikamazumdar@gmail.com

Shyama Prasad Saha, E. mail: shyamaprasad.saha3@gmail.com

Vaskar Das, E.mail: dasvaskar303@gmail.com

[@]Shilpi Ghosh, E.mail: ghosshilpi@gmail.com; shilpighosh@nbu.ac.in

Affiliation: Department of Biotechnology, University of North Bengal, Raja Rammohunpur, P.O.-NBU, Siliguri, West Bengal, India, PIN-734013

*Contributed equally

[@]**Correspondence:** Department of Biotechnology, University of North Bengal, Raja Rammohunpur, Siliguri, West Bengal, India, PIN-734013

E. mail: ghosshilpi@gmail.com

shilpighosh@nbu.ac.in

Genera

Pseudoclostridium	2809	bacteria
Chelatococcus	2129	bacteria
Thermoanaerobacterium	1953	bacteria
Algoriphagus	1379	bacteria
Bacillus	522	bacteria
Pseudoxanthomonas	512	bacteria
Geobacillus	471	bacteria
Halomonas	435	bacteria
Aeribacillus	431	bacteria
Symbiobacterium	234	bacteria
Aneurinibacillus	194	bacteria
Caldibacillus	193	bacteria
Hungateiclostridium	73	bacteria
Vibrio	70	bacteria
Virgibacillus	67	bacteria
Escherichia	63	bacteria
Salmonella	57	bacteria
Micrococcus	53	Actinobacteria
Clostridium	52	bacteria
Tepidimicrobium	49	bacteria
Microvirga	41	bacteria
Parageobacillus	34	bacteria
Halobacillus	33	bacteria
Echinicola	29	bacteria
Anoxybacillus	28	bacteria
Halorubrum	28	archaea
Paenibacillus	26	bacteria
Stenotrophomonas	25	bacteria
Deinococcus	22	bacteria
Bradyrhizobium	20	bacteria
Bosea	19	bacteria
Keratinibaculum	19	bacteria
Corynebacterium	18	Actinobacteria
Thermoanaerobacter	18	bacteria
Methylobacterium	16	bacteria
Rufibacter	16	bacteria
Flavobacterium	15	bacteria
Pseudomonas	15	bacteria
Alcaligenes	14	bacteria
Aquiflexum	14	bacteria
Herbinix	13	bacteria
Cyclobacterium	12	bacteria
Ralstonia	12	bacteria
Xanthomonas	12	bacteria
Belliella	11	bacteria
Paracoccus	11	bacteria
Streptomyces	11	Actinobacteria
Chryseobacterium	10	bacteria
Shigella	10	bacteria

Thermobacillus	10	bacteria
Brachybacterium	9	Actinobacteria
Haloplanus	9	archaea
Microbacterium	9	Actinobacteria
Thermoclostridium	9	bacteria
Chitinophaga	8	bacteria
Desulfotomaculum	8	bacteria
Mesorhizobium	8	bacteria
Methylocaldum	8	bacteria
Caenibacillus	7	bacteria
Caldanaerobius	7	bacteria
Dehalococcoides	7	bacteria
Kocuria	7	Actinobacteria
Luteimonas	7	bacteria
Natronomonas	7	archaea
Sphingomonas	7	bacteria
Thermomonas	7	bacteria
Tuberibacillus	7	bacteria
Agathobaculum	6	bacteria
Citrobacter	6	bacteria
Cupriavidus	6	bacteria
Listeria	6	bacteria
Lutispora	6	bacteria
Natronolimnobius	6	archaea
Silanimonas	6	bacteria
Spirosoma	6	bacteria
Thermoacetogenium	6	bacteria
Achromobacter	5	bacteria
Aquamicrobium	5	bacteria
Arthrobacter	5	Actinobacteria
Brevibacillus	5	bacteria
Cecembia	5	bacteria
Eubacterium	5	bacteria
Mahella	5	bacteria
Mariniradius	5	bacteria
Massilia	5	bacteria
Oceanobacillus	5	bacteria
Rhizobium	5	bacteria
Roseomonas	5	bacteria
Runella	5	bacteria
Ureibacillus	5	bacteria
Acinetobacter	4	bacteria
Acuticoccus	4	bacteria
Amphibacillus	4	bacteria
Anaerobranca	4	bacteria
Azorhizobium	4	bacteria
Azospirillum	4	bacteria
Bordetella	4	bacteria
Burkholderia	4	bacteria
Enterococcus	4	bacteria

Fabibacter	4	bacteria
Halalkaliarchaeum	4	archaea
Halapricum	4	archaea
Hymenobacter	4	bacteria
Inquilinus	4	bacteria
Lysobacter	4	bacteria
Mongoliibacter	4	bacteria
Natronococcus	4	archaea
Owenweeksia	4	bacteria
Pedobacter	4	bacteria
Phreatobacter	4	bacteria
Pontibacter	4	bacteria
Schlegelella	4	bacteria
Sinorhizobium	4	bacteria
Sphingobacterium	4	bacteria
Sporanaerobacter	4	bacteria
Staphyllococcus	4	bacteria
Sulfuritortus	4	bacteria
Variovorax	4	bacteria
Aminobacter	3	bacteria
Anaerosalibacter	3	bacteria
Aureimonas	3	bacteria
Bacteroides	3	bacteria
Bittarella	3	bacteria
Caloramator	3	bacteria
Calorimonas	3	bacteria
Cohnella	3	bacteria
Cutibacterium	3	Actinobacteria
Desulfitibacter	3	bacteria
Desulfofarcimen	3	bacteria
Dyadobacter	3	bacteria
Flavisolibacter	3	bacteria
Geobacter	3	bacteria
Geosporobacter	3	bacteria
Haloarcula	3	archaea
Halobiforma	3	archaea
Halohasta	3	archaea
Halopiger	3	archaea
Lysinibacillus	3	bacteria
Muricauda	3	bacteria
Mycolicibacterium	3	Actinobacteria
Nocardia	3	Actinobacteria
Ochrobactrum	3	bacteria
Paraburkholderia	3	bacteria
Paraflavitalea	3	bacteria
Shinella	3	bacteria
Spiribacter	3	bacteria
Tepidibacter	3	bacteria
Tepidiphilus	3	bacteria
Thalassobacillus	3	bacteria

Thermoactinomyces	3	bacteria
Thermolongibacillus	3	bacteria
Vulcanibacterium	3	bacteria
Xanthobacter	3	bacteria
Zunongwangja	3	bacteria
Acidovorax	2	bacteria
Aeromonas	2	bacteria
Aerosticca	2	bacteria
Afifella	2	bacteria
Alkaliphilus	2	bacteria
Amycolatopsis	2	Actinobacteria
Anaeromyxobacter	2	bacteria
Aquabacter	2	bacteria
Arenibacter	2	bacteria
Arenimonas	2	bacteria
Azoarcus	2	bacteria
Azotobacter	2	bacteria
Bavariicoccus	2	bacteria
Bernardetia	2	bacteria
Bifidobacterium	2	Actinobacteria
Brevibacterium	2	Actinobacteria
Caldimonas	2	bacteria
Camelimonas	2	bacteria
Campylobacter	2	bacteria
Caulobacter	2	bacteria
Chromohalobacter	2	bacteria
Collinsella	2	Actinobacteria
Cytophaga	2	bacteria
Defluviitalea	2	bacteria
Dokdonella	2	bacteria
Dokdonia	2	bacteria
Ectothiorhodospira	2	bacteria
Ensifer	2	bacteria
Epidermidibacterium	2	Actinobacteria
Fusobacterium	2	bacteria
Gottschalkia	2	bacteria
Haemophilus	2	bacteria
Halobacterium	2	archaea
Halobellus	2	archaea
Halomicrobium	2	archaea
Halopenitus	2	archaea
Haloprofundus	2	archaea
Haloquadratum	2	archaea
Halorhabdus	2	archaea
Halorussus	2	archaea
Halostella	2	archaea
Hydrogenophaga	2	bacteria
Klebsiella	2	bacteria
Kordia	2	bacteria
Lactobacillus	2	bacteria

Mucilaginibacter	2	bacteria
Mycobacterium	2	Actinobacteria
Natrialba	2	archaea
Natrinema	2	archaea
Nitratireductor	2	bacteria
Nocardioides	2	Actinobacteria
Nostoc	2	bacteria
Pannonibacter	2	bacteria
Pantoea	2	bacteria
Paramaledivibacter	2	bacteria
Peptoniphilus	2	bacteria
Petrotoga	2	bacteria
Pseudarcicella	2	bacteria
Pseudorhodoferax	2	bacteria
Quasibacillus	2	bacteria
Rhodanobacter	2	bacteria
Rhodopseudomonas	2	bacteria
Roseovarius	2	bacteria
Rubrivivax	2	bacteria
Ruminococcus	2	bacteria
Salarchaeum	2	archaea
Sediminicola	2	bacteria
Sphingopyxis	2	bacteria
Stappia	2	bacteria
Streptacidiphilus	2	Actinobacteria
Streptococcus	2	bacteria
Sulfurivermis	2	bacteria
Syntrophomonas	2	bacteria
Tepidanaerobacter	2	bacteria
Thermaerobacter	2	bacteria
Thioalkalivibrio	2	bacteria
Vogesella	2	bacteria
Westiellopsis	2	bacteria
Zhizhongheella	2	bacteria
Actinobaculum	1	Actinobacteria
Actinomadura	1	Actinobacteria
Actinopolyspora	1	Actinobacteria
Agrobacterium	1	bacteria
Aidingimonas	1	bacteria
Alcanivorax	1	bacteria
Algibacter	1	bacteria
Alicyclobacillus	1	bacteria
Alkalibacillus	1	bacteria
Altererythrobacter	1	bacteria
Anaerobacterium	1	bacteria
Anaerocolumna	1	bacteria
Anaerophaga	1	bacteria
Anaerosacchariphilus	1	bacteria
Andreprevotia	1	bacteria
Aphanothece	1	bacteria

Aquimarina	1	bacteria
Arachidicoccus	1	bacteria
Aulosira	1	bacteria
Aurantimonas	1	bacteria
Auraticoccus	1	Actinobacteria
Baekduia	1	Actinobacteria
Beggiatoa	1	bacteria
Belnapia	1	bacteria
Borrelia	1	bacteria
Brachyspira	1	bacteria
Brockia	1	bacteria
Caballeronia	1	bacteria
Caldalkalibacillus	1	bacteria
Caldicoprobacter	1	bacteria
Caloranaerobacter	1	bacteria
Calothrix	1	bacteria
Caproiciproducens	1	bacteria
Carboxydothermus	1	bacteria
Castellaniella	1	bacteria
Coprococcus	1	bacteria
Cellulophaga	1	bacteria
Cellulosilyticum	1	bacteria
Cerasibacillus	1	bacteria
Chelativorans	1	bacteria
Chlorobaculum	1	bacteria
Chromobacterium	1	bacteria
Clostridiisalibacter	1	bacteria
Clostridioides	1	bacteria
Colwellia	1	bacteria
Cronobacter	1	bacteria
Cyanobium	1	bacteria
Cystobacter	1	bacteria
Dechlorosoma	1	bacteria
Defluviitoga	1	bacteria
Dehalobacterium	1	bacteria
Delftia	1	bacteria
Desnuesiella	1	bacteria
Desulfotomaculum	1	bacteria
Desulfofustis	1	bacteria
Desulfohalotomaculum	1	bacteria
Desulfosporosinus	1	bacteria
Duncaniella	1	bacteria
Dyella	1	bacteria
Edaphobacter	1	bacteria
Egicoccus	1	Actinobacteria
Eisenibacter	1	bacteria
Elioraea	1	bacteria
Empedobacter	1	bacteria
Enterovirga	1	bacteria
Ethanoligenens	1	bacteria

Euzebyella	1	bacteria
Faecalibacterium	1	bacteria
Falsochrobactrum	1	bacteria
Ferruginivarius	1	bacteria
Fervidicella	1	bacteria
Fervidicola	1	bacteria
Flavipsychrobacter	1	bacteria
Fluviicola	1	bacteria
Fonticella	1	bacteria
Fontimonas	1	bacteria
Frankia	1	Actinobacteria
Fransicella	1	bacteria
Fulvivirga	1	bacteria
Gaetbulibacter	1	bacteria
Gemmatirosa	1	bacteria
Geomonas	1	bacteria
Gracilibacillus	1	bacteria
Gramella	1	bacteria
Granulibacter	1	bacteria
Halalkalicoccus	1	archaea
Halanaeroarchaeum	1	archaea
Haliscomenobacter	1	bacteria
Haloarchaeobius	1	archaea
Halococcus	1	archaea
Halodesulfurarchaeum	1	archaea
Haloferax	1	archaea
Halogeometricum	1	archaea
Halogranum	1	archaea
Halomarina	1	archaea
Halomicroarcula	1	archaea
Halonotius	1	archaea
Haloterrigena	1	archaea
Hartmannibacter	1	bacteria
Heliobacterium	1	bacteria
Herbaspirillum	1	bacteria
Herminiimonas	1	bacteria
Hespellia	1	bacteria
Hoeflea	1	bacteria
Ideonella	1	bacteria
Indibacter	1	bacteria
Isoptericola	1	Actinobacteria
Izhakiella	1	bacteria
Kineosphaera	1	Actinobacteria
Kribbia	1	Actinobacteria
Labilibaculum	1	bacteria
Laceyella	1	bacteria
Lawsonibacter	1	bacteria
Leclercia	1	bacteria
Legionella	1	bacteria
Lentimicrobium	1	bacteria

Leptobacterium	1	bacteria
Leptospirillum	1	bacteria
Lyngbya	1	bacteria
Macrococcus	1	bacteria
Magnetospirillum	1	bacteria
Mangrovibacter	1	bacteria
Mannheimia	1	bacteria
Maribacter	1	bacteria
Marichromatium	1	bacteria
Marinagarivorans	1	bacteria
Marinirhabdus	1	bacteria
Marinitoga	1	bacteria
Marinococcus	1	bacteria
Marispirochaeta	1	bacteria
Martelella	1	bacteria
Massilibacterium	1	bacteria
Methanoregula	1	archaea
Methylacidophilum	1	bacteria
Methylococcus	1	bacteria
Methylocystis	1	bacteria
Methylomusa	1	bacteria
Methylorubrum	1	bacteria
Methyloversatilis	1	bacteria
Microbispora	1	Actinobacteria
Microcystis	1	bacteria
Microlunatus	1	Actinobacteria
Micromonospora	1	Actinobacteria
Minicystis	1	bacteria
Moheibacter	1	bacteria
Mongoliimonas	1	bacteria
Myroides	1	bacteria
Natronobacterium	1	archaea
Natronorubrum	1	archaea
Neisseria	1	bacteria
Neorhizobium	1	bacteria
Nibribacter	1	bacteria
Nitratifractor	1	bacteria
Nitrococcus	1	bacteria
Nitrosococcus	1	bacteria
Nonomuraea	1	Actinobacteria
Novibacillus	1	bacteria
Noviherbaspirillum	1	bacteria
Oceanithermus	1	bacteria
Opitutus	1	bacteria
Ornithinibacillus	1	bacteria
Paenisporosarcina	1	bacteria
Paludifilum	1	bacteria
Pandoraea	1	bacteria
Paraferrimonas	1	bacteria
Paramesorhizobium	1	bacteria

Pararhizobium	1	bacteria
Phyllobacterium	1	bacteria
Planococcus	1	bacteria
Planomicrobium	1	bacteria
Pleomorphomonas	1	bacteria
Polaromonas	1	bacteria
Polymorphum	1	bacteria
Polynucleobacter	1	bacteria
Pontibacillus	1	bacteria
Pradoshia	1	bacteria
Prevotella	1	bacteria
Prochlorococcus	1	bacteria
Prosthecochloris	1	bacteria
Proteiniborus	1	bacteria
Proteus	1	bacteria
Pseudoalteromonas	1	bacteria
Pseudobacteroides	1	bacteria
Pseudooceanicola	1	bacteria
Pseudorhizobium	1	bacteria
Ramlibacter	1	bacteria
Raoultella	1	bacteria
Reyranella	1	bacteria
Rhodobacter	1	bacteria
Rhodoblastus	1	bacteria
Rhodocytophaga	1	bacteria
Rhodoligotrophos	1	bacteria
Rhodonellum	1	bacteria
Rhodoplanes	1	bacteria
Rhodovulum	1	bacteria
Robiginitalea	1	bacteria
Roseateles	1	bacteria
Roseivirga	1	bacteria
Rubidibacter	1	bacteria
Rubrimonas	1	bacteria
Rubrivorax	1	bacteria
Saccharibacillus	1	bacteria
Saccharothrix	1	bacteria
Salicibibacter	1	bacteria
Salinarimonas	1	bacteria
Salinibacter	1	bacteria
Salinimicrobium	1	bacteria
Salinispira	1	bacteria
Salipaludibacillus	1	bacteria
Salipiger	1	bacteria
Sandaracinus	1	bacteria
Saprospira	1	bacteria
Schleiferia	1	bacteria
Scytonema	1	bacteria
Selenomonas	1	bacteria
Seonamhaeicola	1	bacteria

Serinicoccus	1	Actinobacteria
Serpentinomonas	1	bacteria
Serratia	1	bacteria
Shewanella	1	bacteria
Singulisphaera	1	bacteria
Sinohodobacter	1	bacteria
Solirubrobacter	1	Actinobacteria
Sorangium	1	bacteria
Sphingobium	1	bacteria
Stanieria	1	bacteria
Staphylospora	1	bacteria
Stella	1	bacteria
Streptosporangium	1	Actinobacteria
Tepidicaulis	1	bacteria
Tepidicella	1	bacteria
Tepidimonas	1	bacteria
Thalassococcus	1	bacteria
Thauera	1	bacteria
Thermococcus	1	archaea
Thermohalobacter	1	bacteria
Thermosediminibacter	1	bacteria
Thermovenabulum	1	bacteria
Thioclava	1	bacteria
Tissierella	1	bacteria
Tistrella	1	bacteria
Treponema	1	bacteria
Trinickia	1	bacteria
Ureaplasma	1	bacteria
Vallitalea	1	bacteria
Verticiella	1	bacteria
Winogradskyella	1	bacteria
Xylanibacterium	1	Actinobacteria
Zobellella	1	bacteria
Zobiella	1	bacteria
Total genus	13191	
No significant similarity	2243	unclassified
deleted (contamination)	29	unclassified
Uncultured firmicutes bacterium contig	1	unclassified
Uncultured bacterium clone	1	unclassified
Uncultured bacterium	3	unclassified
Uncultured firmicutes bacterium contig _31	1	unclassified
Uncultured prokaryote clone	1	unclassified
Bacterium 0.1xD8-71	1	unclassified
bacterium 1xD42-11	1	unclassified
Bacterium 1xD42-62	1	unclassified
bacterium D16-76	3	unclassified
bacterium M00.F.Ca.ET.177.01.1.1	1	unclassified
Halophilic archaeon	2	unclassified
Candidatus Pelagibacter sp	1	unclassified

PREDICTED: Cicer arietinum	1	unclassified
PREDICTED: Cucumis melo	1	unclassified
PREDICTED: Drosophila erecta	1	unclassified
PREDICTED: Ethiostoma	1	unclassified
PREDICTED: Lactuca sativa	1	unclassified
PREDICTED: Lingula anatina	1	unclassified
PREDICTED:Loxodonta africana	1	unclassified
PREDICTED:Panicum hali	1	unclassified
PREDICTED: Pteropus alecto	1	unclassified
PREDICTED: Stomoxys calcitrans	1	unclassified
PREDICTED: Vanessa tameamea	1	unclassified
Bacillaceae bacterium	17	unclassified
Balneolaceae bacterium	1	unclassified
Chromatiaceae bacterium	1	unclassified
Clostridiales bacterium	1	unclassified
Corynebacteriaceae bacterium	5	unclassified
Cytophagales bacterium	1	unclassified
Enterobacteriaceae bacterium	1	unclassified
Enterococcaceae bacterium	1	unclassified
Erysipelotrichaceae bacterium	1	unclassified
Flavobacteriaceae bacterium	1	unclassified
Gemmataceae bacterium	1	unclassified
Phycisphaerae bacterium	1	unclassified
Planctomyecetes bacterium	5	unclassified
Prolixibacteraceae bacterium	1	unclassified
Rhodanobacteriaceae bacterium	1	unclassified
Rhodocyclaceae bacterium	5	unclassified
Ruminococcaceae bacterium	2	unclassified
Sterolibacteriaceae bacterium	2	unclassified
Streptococcaceae bacterium	1	unclassified
Thermoanaerobacteraceae bacterium	4	unclassified
Halobacteriaceae archaeon	4	unclassified
Aquilla chrysaetos chrysaetos	1	eagle
Aphantopus	1	insect
Mus	2	rat
Caligus	1	sea lice
Homo sapiens	2	man
Arabia	1	not found
Cyphellophora	1	fungi
Cryptococcus	1	fungi
Eremothecium	1	fungi
Exophiala	1	fungi
Lachancea	2	fungi
Sciurus	1	fungi
Streptopelia	1	fungi
Ustillago	1	fungi
Candida glabrata	55	fungi
Cladophialophora	1	fungi
Oryza sativa Indica group cultivar	3	rice
Gossypium	1	cotton

Triticum	2	wheat
Vigna	1	plant
Digitaria	1	plant
Cyprinus	1	fish
Salmo	1	fish
Strongyloides	1	worm
